# Supplementary material for: The GEF Trio controls endothelial cell size and arterial remodeling downstream of Vegf signaling in both zebrafish and cell models
Source: Nat Commun. 2020 Oct 21;11:5319. doi: 10.1038/s41467-020-19008-0 (PMC7578835; doi:10.1038/s41467-020-19008-0)
Supplement: Supplementary file 1 — Supplementary Information [file 41467_2020_19008_MOESM1_ESM.pdf]

The GEF Trio controls endothelial cell size and arterial remodeling downstream of Vegf in both zebrafish and cell models

Klems et al.

Supplementary Figure 1

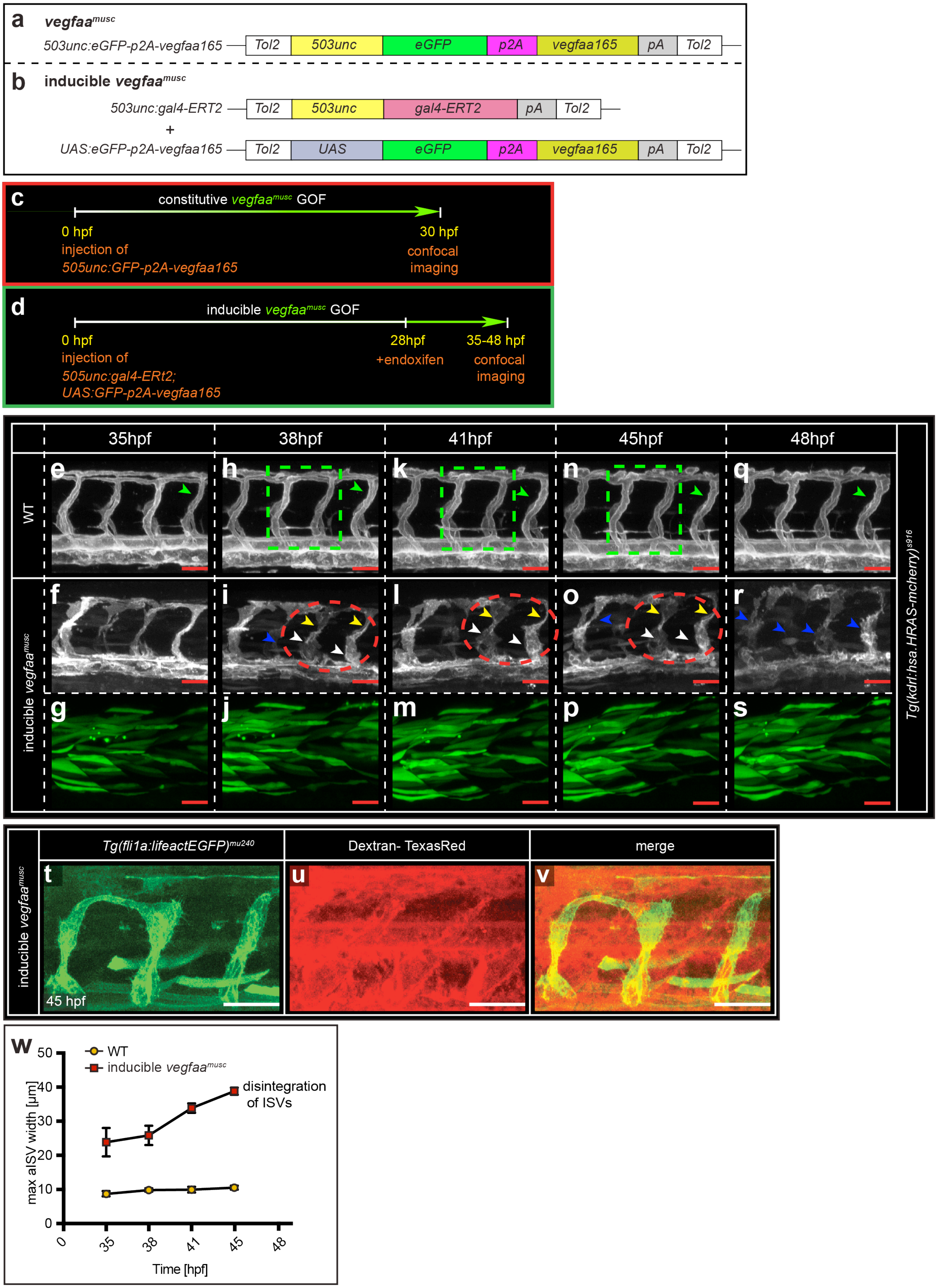

Supplementary Figure 1: *Vegfaa* constitutive and inducible gain of function models.

(**A,B**) Structure of the Tol2 constructs used for muscle-specific overexpression of *vegfaa165* in a constitutive (*vegfaa*^{musc}, A) and in an inducible manner (inducible *vegfaa*^{musc}, B). (A) For *vegfaa*^{musc}, the muscle promoter *503unc* drives *vegfaa165* tagged to *eGFP* via the self-cleaving peptide p2A. (B) For inducible *vegfaa*^{musc}, *503unc* drives the endoxifen-inducible *gal4-ERT2*. *vegfaa165* tagged to *eGFP-p2A* is under the control of the *UAS* promoter.

(**C,D**) The experimental protocol for the constitutive (C) and inducible (D) *vegfaa* gain of function approach. For the constitutive approach, imaging was performed at 30hpf and not continued at later stages as the vasculature was already severely disrupted at this stage. For the inducible approach, endoxifen was added at 28hpf, and confocal imaging started at 35hpf.

(**E,H,K,N,Q**) *In vivo* confocal imaging of aISV in developing WT embryos at indicated time points. Green box and arrowhead: note well organized and lumenized aISVs in WT. Images are representative for n=3 independent embryos per scenario. Scale bar, 50µm.

(**F,I,L,O,R**) *In vivo* confocal imaging of aISV in developing inducible *vegfaa*^{musc} embryos at indicated time points. Transgene was induced at 28hpf, imaging started at 35hpf. Images are representative for n=4 independent embryos. Note that at 35hpf some vessels already showed signs of enlargement. Blue arrowheads indicate disintegrating vessels. Red circle depicts two aISVs; the white arrowheads show an enlarged dorsal part; the yellow arrowheads show a smaller/constricted part. The indicated vessels were not functional. Scale bar, 50µm.

(**G,J,M,P,S**) *In vivo* imaging of the transgene expression (*vegfaa-p2a-GFP*) confirms expression in developing muscle tissue (*503unc* is a muscle specific promoter). Images are representative for n=4 embryos. Scale bar, 50µm.

(**T-V**) *In vivo* confocal imaging of plasma extravasation in inducible *vegfaa* gain of function embryo after injection of 70kDa Dextran-Texas Red. Note the aberrant morphology of aISV (T) and extensive plasma extravasation (U). Images are representative for n=7 independent embryos. Scale bar, 50µm.

(**W**) Quantification of the maximum arterial ISV width dimensions in WT and inducible *vegfaa*^{musc} embryos. As most enlarged aISVs in inducible *vegfaa*^{musc} embryos were not functional and were not carrying flow, we termed the dimension - max width, see white arrowheads in (I,L,O) – and not diameter, solely to indicate this fact. The observations show that while some parts of the vessels are enlarged compatible with Vegf promoting growth, the vasculature is non-functional and disintegrates with time rendering this inducible *vegfaa* approach not useful for targeting arterial diameter. Mean±s.e.m., n=4 independent embryos per indicated genotype.

Supplementary Figure 2

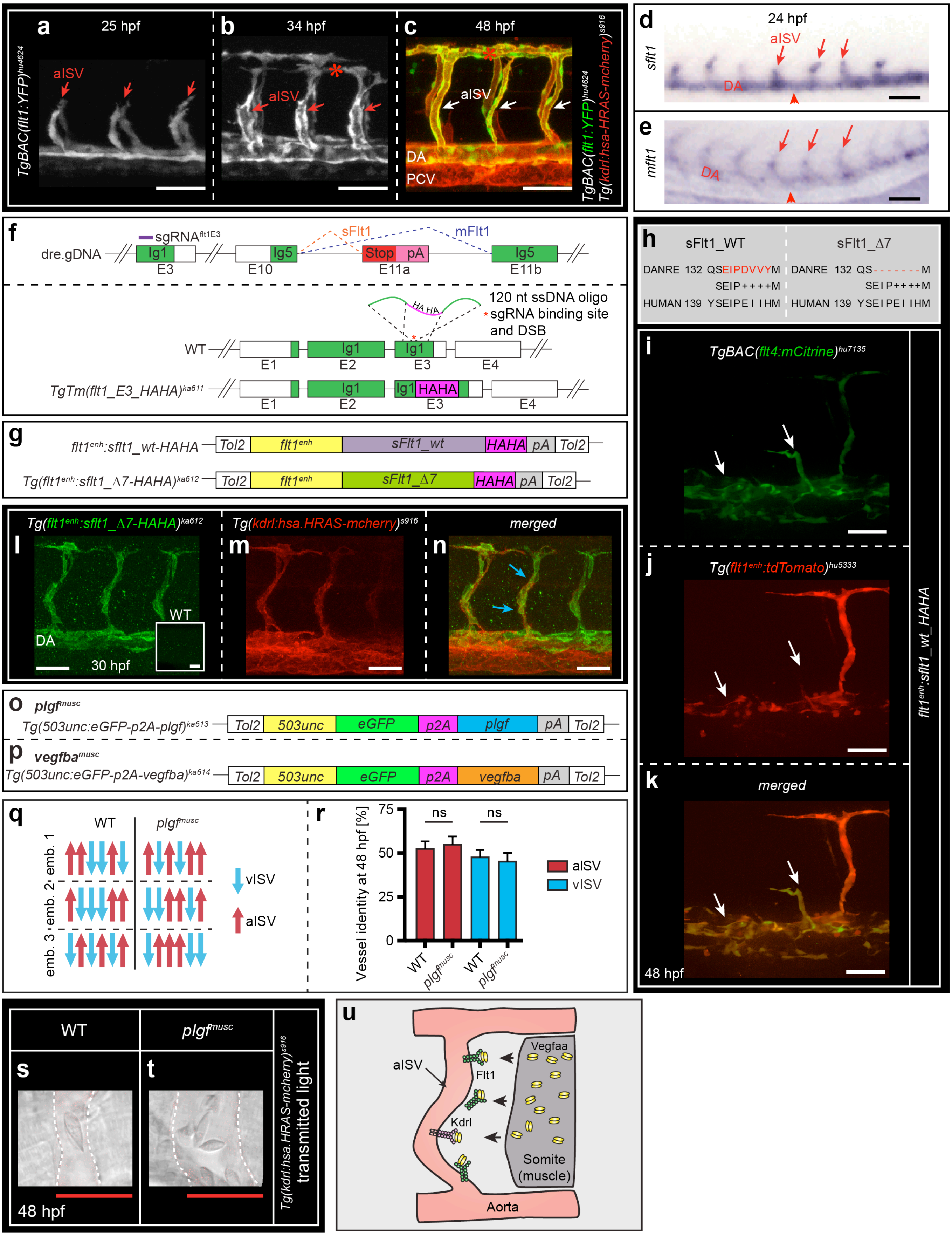

Supplementary Figure 2: Approach for generating knockin and HA-tagged *flt1* transgenics.

(**A-C**) *In vivo* imaging of the trunk vasculature in *TgBAC(flt1:YFP)^{hu4624};Tg(kdrl:hsa-HRAS-mCherry)^{s916}* embryos at indicated time points. Arrows indicate developing arterial intersegmental vessel (aISV); Asterisk indicates the dorsal longitudinal anastomotic vessel (DLAV). DA, dorsal aorta; PCV, posterior cardinal vein. Images represent n=20 examined independent embryos.
(**D,E**) Whole mount *in situ* hybridization for *sflt1* (D) and *mflt1* (E); both isoforms are expressed in aISVs (arrows) and the dorsal aorta (DA, arrowheads). Images are representative for n=19,16 embryos per condition examined over 3 separate experiments.
(**F**) Flt1 exon structure and splice sites for zebrafish *sflt1* and *mflt1*. Domain targeted by sgRNA in exon 3 (*full flt1* mutant *flt1^{ka601}*, targeting both *sflt1* and *mflt1*) is indicated (upper panel).
Strategy for generating the Flt1-HAHA tag knock-in transgenic *TgTm(flt1_E3_HAHA)^{ka611}*: with a CRISPR/Cas9 approach, using sgRNAs directed to *flt1* exon 3, a double-strand break (DSB) was introduced. A 120 nucleotide, single stranded DNA oligonucleotide containing two HA tags, flanked by two homology arms complementary to the region of exon 3 flanking the DSB, was used to introduce the HAHA tag in frame with the *flt1* coding sequence (lower panel).
(**G**) Composition of the Tol2 constructs used for generating *flt1^{enh}:sflt1_wt-HAHA* (top panel) and *Tg(flt1^{enh}:sflt1_Δ7-HAHA)^{ka612}* (bottom panel). The aISV specific *flt1^{enh}* promoter drives expression of either WT *sflt1* or a mutant *sflt1* form (*Δ7)* which is devoid of the Vegfaa binding domain, coupled to two HA tags.
(**H**) Comparison of the Vegf binding site of sFlt1 in Human and Danio rerio; domain crucial for Vegf binding indicated in red. Amino acid sequence of indicated region of sFlt1_WT (left panel) and sFlt1_Δ7 (right panel). The amino acids corresponding to the Vegf binding domain (labeled red) were deleted to obtain sFlt1_Δ7.
(**I-K**) Compromised ISV formation (white arrows) upon expression of *sflt1_wt-HAHA* under control of the *flt1^{enh}* promoter; Flt1-HAHA is functional, and the phenotype compatible with sFlt1-HAHA acting as a Vegfaa scavenger, thereby reducing Vegfaa bio-availability, Kdrl signaling and ISV development. Images represent n=27 embryos examined over 2 independent experiments.
(**L-N**) Whole mount immunostaining with anti-HA antibody showing sFlt1 protein distribution in *Tg(kdrl:hsa.HRAS-mcherry)^{s916}*; *Tg(flt1^{enh}:sflt1_Δ7-HAHA)^{ka612}* double transgenic embryos (n=45 embryos derived from 3 independent experiments). aISVs and DA express sFlt1 protein. The white squared inset in (L) indicates control staining in WT embryos.
(**O,P**) Composition of the Tol2 constructs used for generating *Tg(503unc:eGFP-p2A-plgf)^{ka613}* here referred to as *plgf^{musc}* (O), and *Tg(503unc:eGFP-p2A-vegfba)^{ka614}* here referred to as *vegfba^{musc}* (P).
(**Q,R**) Perfusion characteristics of trunk ISVs of three WT and three *plgf^{musc}* gain of function embryos at 2 dpf (Q), and quantification of arterial-venous vessel identity upon *plgf^{musc}* gain of function (R); n=25,28 embryos per indicated genotype analysed over 3 separate experiments. Arrows indicate flow direction, red is aISV, blue is vISV. All ISVs were perfused, *plgf^{musc}* gain of function had no effect on AV-identity of ISVs. Mean±s.e.m, unpaired two-sided students *t*-test. ns, not significant.
(**S,T**) Imaging of red blood cell perfusion in WT (S) and *plgf^{musc}* (T). The aISVs in *plgf^{musc}* embryos carry blood flow; note in *plgf^{musc}* that the aISV is enlarged and able to carry multiple erythrocytes, as opposed to a single erythrocyte typically passing in the WT aISV scenario. Images are representative for n=52,51 aISVs from 10 embryos per genotype examined over 2 separate experiments. (**U**) Schematic representation of Flt1, Vegfaa and Kdrl distribution in the trunk. Vegfaa produced in the somites (grey area) diffuses towards the aISV where it binds to arterial Flt1. Arterial Flt1 locks Vegfaa in close proximity to Kdrl expressing arterial endothelial cells. Arterial Flt1 acts as a rheostat determining Vegfaa bio-availability for arterial Kdrl receptors.

Scale bar, 50 µm in A-E, I-N; 20 µm in S,T, aISV, arterial intersegmental vessel; DA, dorsal aorta; PCV, posterior cardinal vein; DSB, double-strand break; E1-4, exon 1-4; hpf, hours post fertilization; pA, poly-A tail.

Supplementary Figure 3

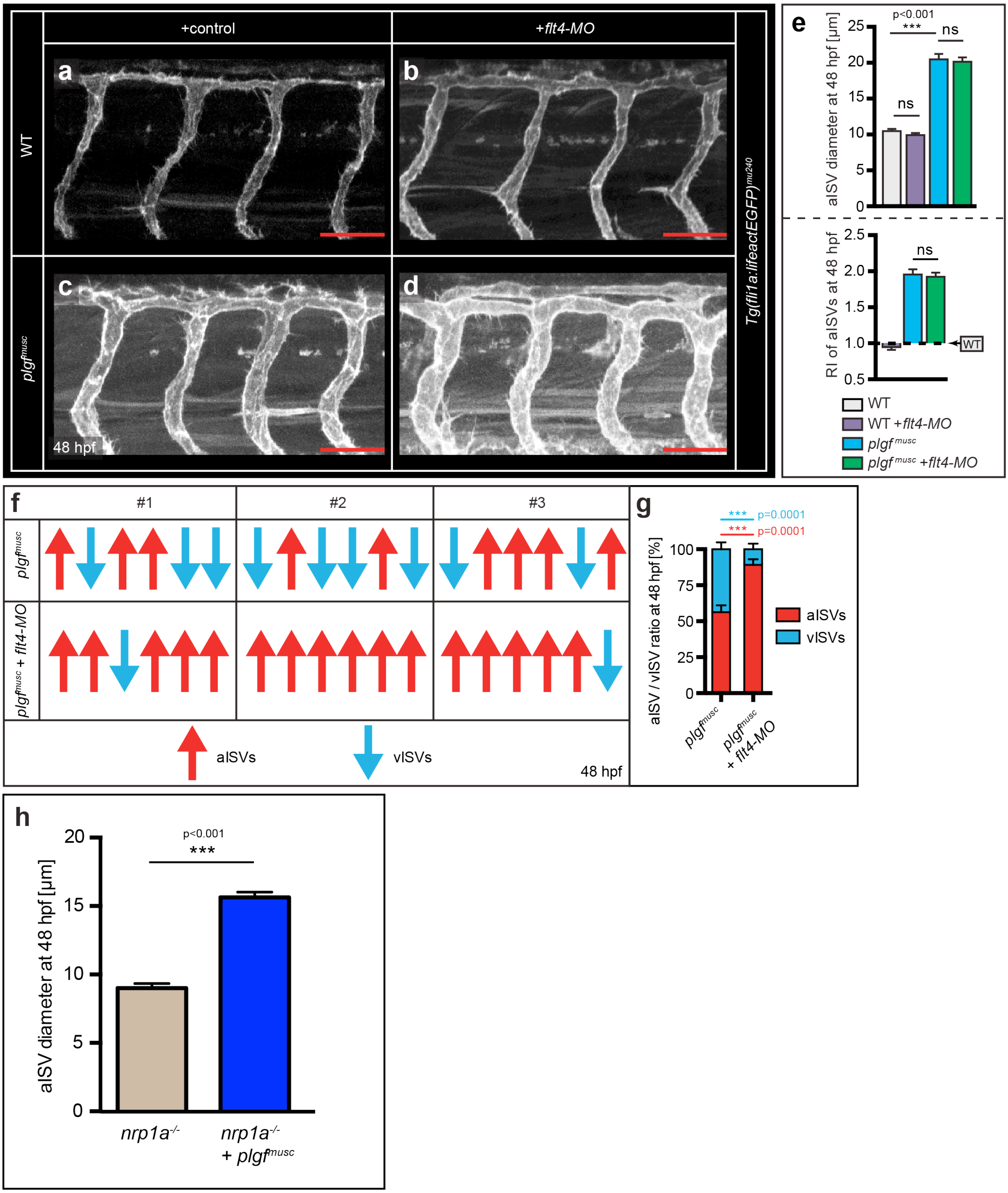

Supplementary Figure 3: *flt4* is not required for Plgf induced arterial outward remodeling

(**A,B**) *In vivo* confocal imaging of aISV in WT embryo injected with control morpholino (A) or *flt4* targeting morpholino (B). Images represent n=27,34 embryos per indicated condition derived from 3 independent experiments. Scale bar, 50µm.

(**C,D**) *In vivo* confocal imaging of aISVs in $plgf^{musc}$ embryo injected with control morpholino (C), or *flt4* targeting morpholino (D). Images represent n=33,32 embryos per indicated condition derived from 3 separate experiments. Scale bar, 50µm.

(**E**) Diameter quantification of images in A-D. Lower panel shows the remodeling index (RI). Mean±s.e.m, one-way ANOVA & posthoc bonferroni test, n=13,20,28,35 aISVs for indicated scenarios. ns, not significant, ***p<0.001.

(**F**) Perfusion characteristics of trunk ISVs at 2dpf, of three $plgf^{musc}$ embryos, and three $plgf^{musc}$ embryos injected with *flt4* targeting morpholino. Arrows indicate flow direction, red is aISV, blue is vISV. All ISVs were perfused.

(**G**) Quantification of arterial-venous vessel identity in $plgf^{musc}$, and in $plgf^{musc}$ injected with *flt4* targeting morpholino. Red is aISV, blue is vISV. Note a significant shift in the aISV/vISV ratio from about 50:50 to 96:4. n=18 embryos per group examined over 3 independent experiments, mean±s.e.m., unpaired two-sided students *t*-test, ***p=0.0001.

(**H**) Quantification of Plgf induced aISV diameter growth in $nrp1a^{-/-}$ mutants. n=65,71 aISVs from 14 embryos per group examined over 2 separate experiments, mean±s.e.m, unpaired two-sided students *t*-test, ***p<0.001.

Supplementary Figure 4

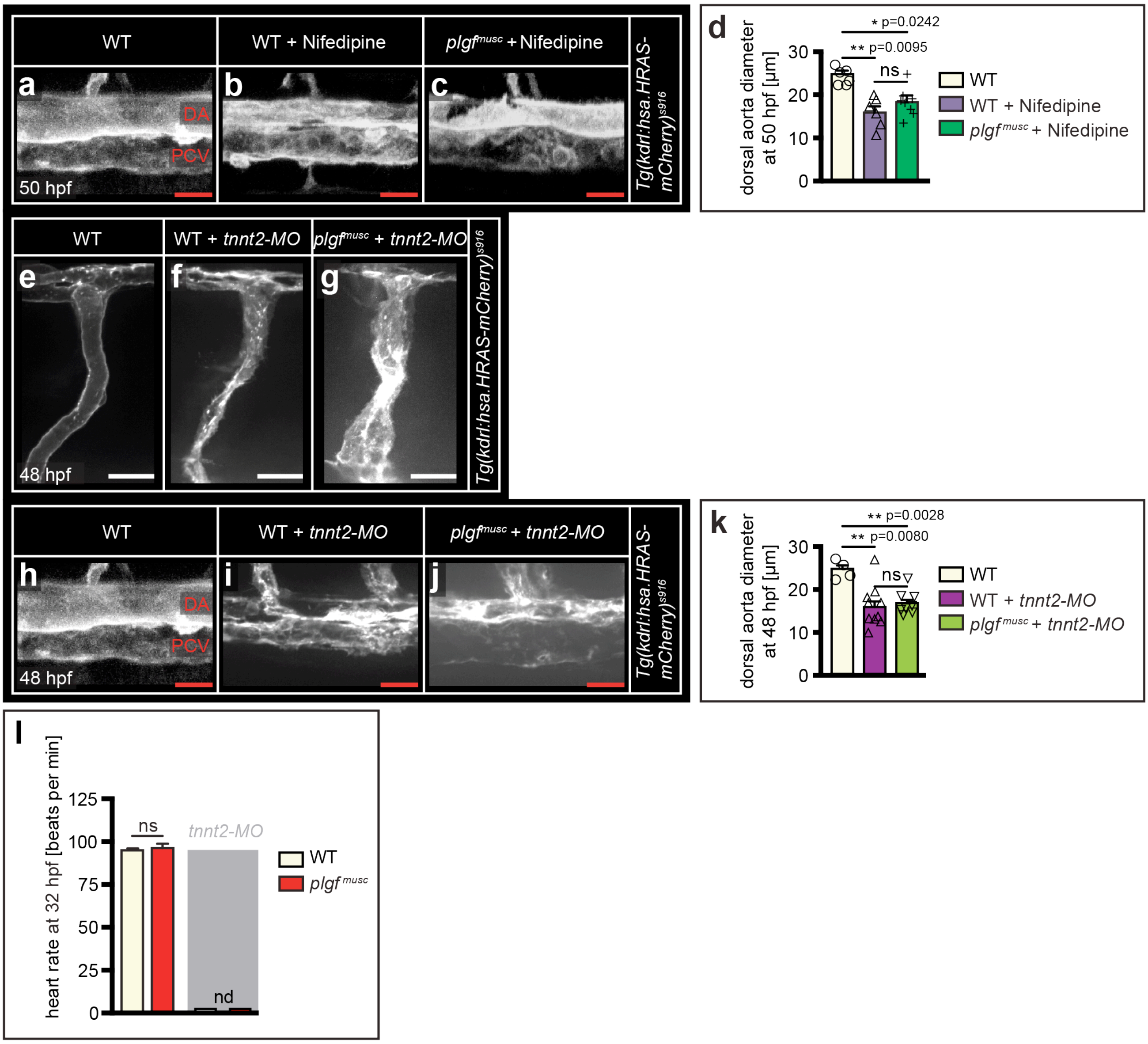

Supplementary Figure 4: Plgf and arterial remodeling during low flow conditions.

(**A-C**) *In vivo* confocal imaging of the dorsal aorta in WT (A), WT treated with nifedipine (B), and *plgf*^{musc} treated with nifedipine (C). Images are representative for n=24,27,26 animals per indicated group derived from 3 separate experiments. Scale bar, 25 μm.

(**D**) Quantification of images in A-C. Mean±s.e.m, unpaired two-sided students *t*-test, n=6,6,8 embryos per group. ns, not significant, *p=0.0242, **p=0.0095.

(**E-G**) *In vivo* confocal imaging of aISV in WT (E), WT injected with *tnnt2* targeting morpholino (F), *plgf*^{musc} injected with *tnnt2* targeting morpholino (G). Images are representative for n=68,73,72 aISVs per indicated scenario examined over 3 independent experiments. Scale bar, 25 μm.

(**H-J**) *In vivo* confocal imaging of the dorsal aorta in WT (H), WT injected with *tnnt2* targeting morpholino (I), and *plgf*^{musc} injected with *tnnt2* targeting morpholino (J). Images represent n=25,23,25 animals per indicated group derived from 3 separate experiments. Scale bar, 25 μm.

(**K**) Quantification of images in H-J. Mean±s.e.m, unpaired two-sided students *t*-test, n=4,10,9 embryos per group, ns, not significant, **p<0.01.

(**L**) Heart rate in WT and *plgf*^{musc} embryos, and embryos injected with *tnnt2* targeting morpholino. Heart rate was not detectable in the latter scenario. Mean±s.e.m, unpaired two-sided students *t*-test, n=30,30,20,20 embryos per indicated group. nd, not detectable. ns, not significant.

Supplementary Figure 5

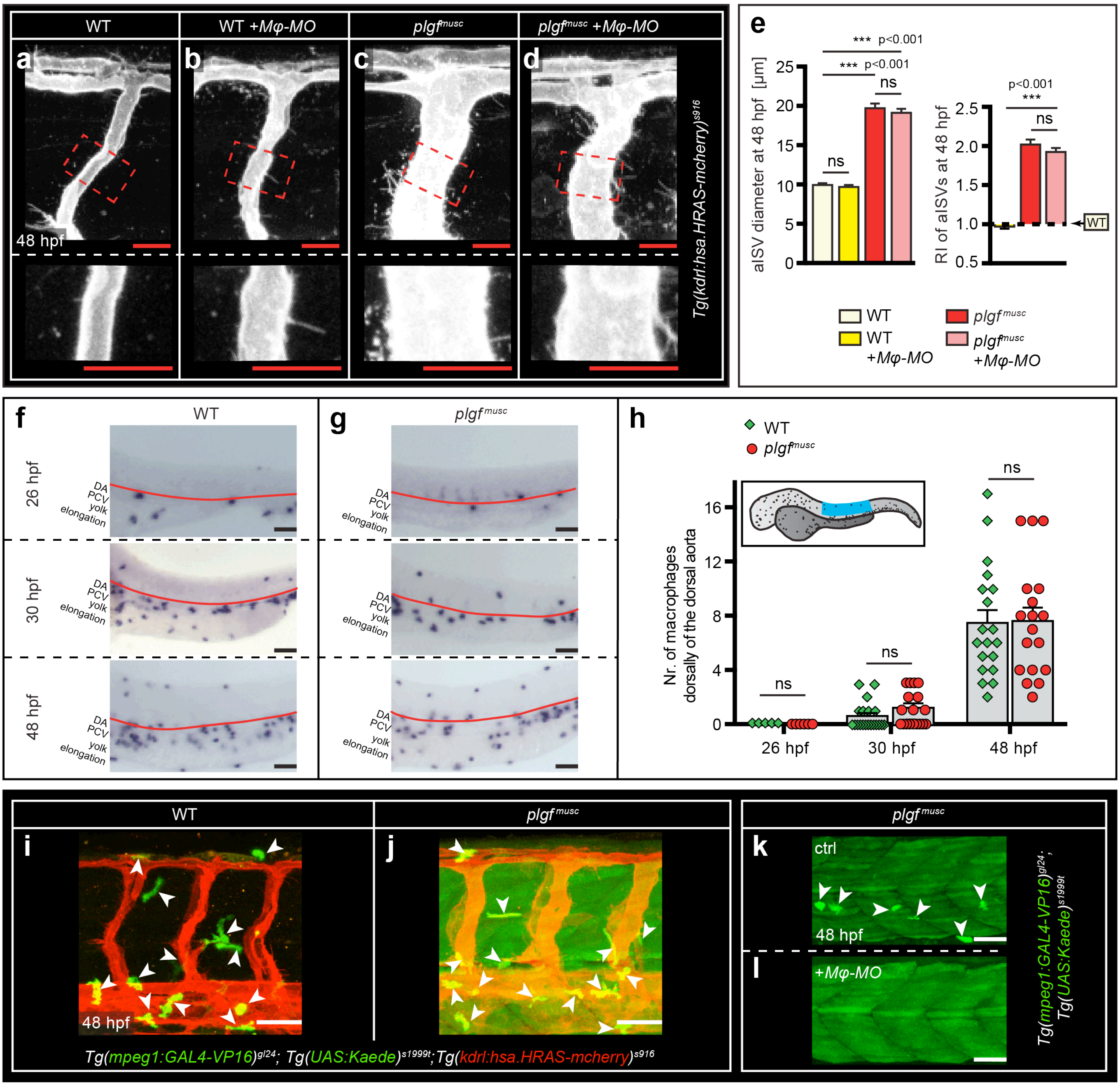

Supplementary Figure 5: Macrophages are not required for Plgf induced outward arterial remodeling.

(**A-D**) *In vivo* confocal imaging of aISV in WT embryo (A), WT injected with 3 morpholinos, targeting *pu.1*, *gcsfr* and *irf8* to reduce macrophage differentiation (B), *plgf^{musc}* embryos (C), and *plgf^{musc}* upon morpholino knockdown of *pu.1*, *gcsfr* and *irf8* (D). Red dotted box is displayed at higher magnification in the lower panel. Images represent n=36,52,49,61 aISVs per group examined from 3 independent experiments. Scale bar, 25 μm.

(**E**) Diameter quantification of images in A-D; mean±s.e.m, unpaired two-sided students *t*-test, n=19,30,33,32 aISVs per indicated group. Note: unaltered arterial diameter growth upon reduced macrophage differentiation. Right panel shows the remodeling index (% change versus WT with WT=1.0). ns, not significant, ***p<0.001.

(**F,G**) Whole mount *in situ* hybridization with the macrophage marker *l-plastin* showing macrophage distribution in the trunk of WT (F) and *plgf^{musc}* embryo (G) at indicated time points. Images are representative for n=38 embryos per scenario derived from 3 independent experiments. Scale bar, 25 μm.

(**H**) Quantification of macrophage distribution. Mean±s.e.m, unpaired two-sided students *t*-test, n=5,6 embryos per genotype at 26 hpf, n=20 embryos per genotype at 30 hpf, n=19,18 embryos per genotype at 48 hpf. ns, not significant.

(**I,J**) *In vivo* imaging of macrophages in *Tg(mpeg1:GAL4-VP16)^{gl24}; Tg(UAS:Kaede)^{s1999t}; Tg(kdrl:hsa.HRAS-mcherry)^{s916}* in WT (I) and *plgf^{musc}* embryos (J). Arrowheads indicate macrophages. Note: the slight green background on the muscle tissue is due to the eGFP linked to *plgf* via p2A. Images are representative for n=28,36 embryos per genotype examined over 3 separate experiments. Scale bar, 25 μm.

(**K,L**) *In vivo* imaging of macrophages in *plgf^{musc}* injected with control morpholino (K) and after inhibition of macrophage differentiation using a combination of *pu.1*, *gcsfr* and *irf8* targeting morpholinos (L). Arrowheads indicate macrophages. Note reduced macrophages in (L). Images represent n=33,38 animals per group examined from 3 separate experiments. Scale bar, 50 μm.

aISV, arterial intersegmental vessel; DA, dorsal aorta; PCV, posterior cardinal vein; hpf, hours post fertilization; RI, remodeling index; MO, morpholino.

Supplementary Figure 6

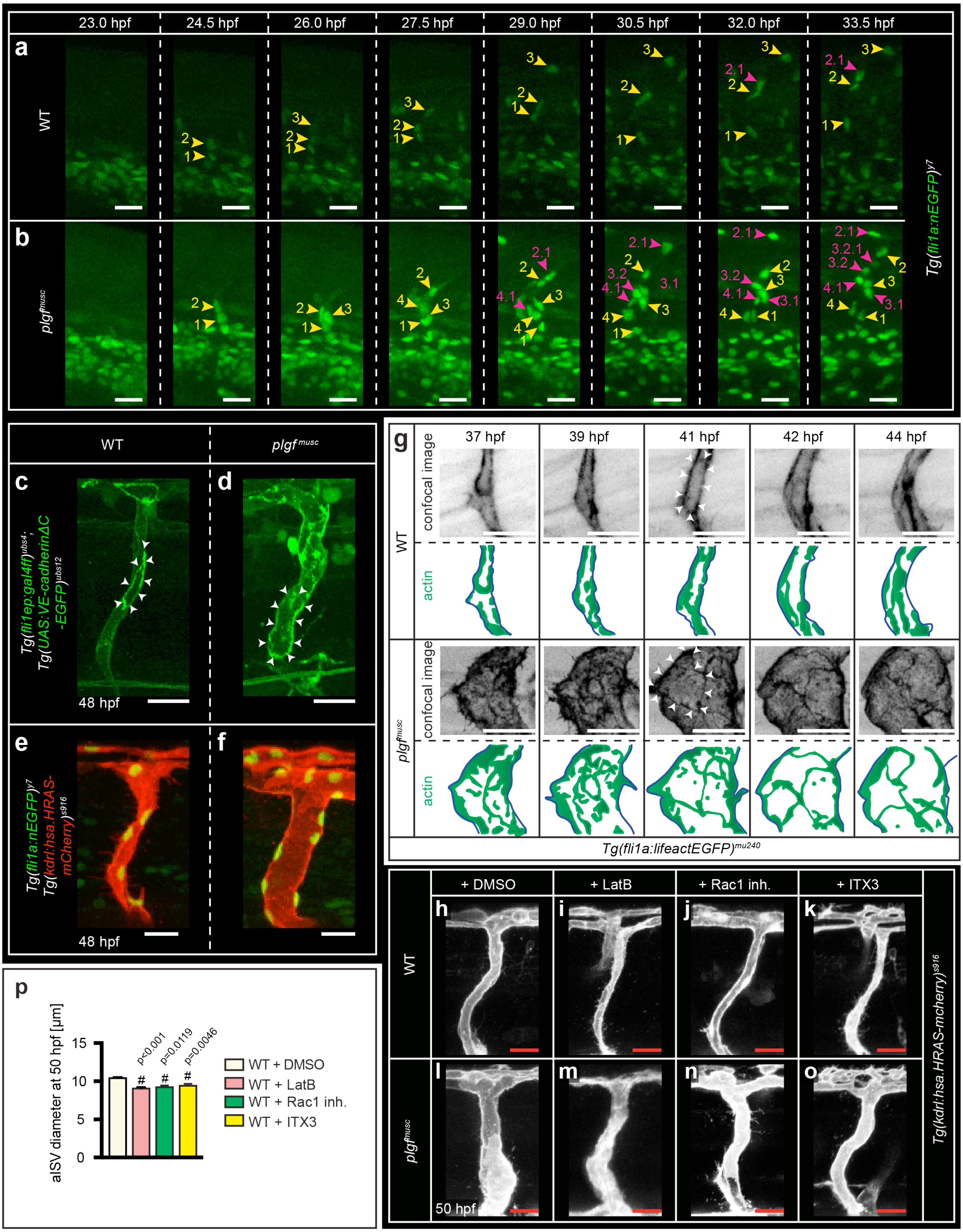

Supplementary Figure 6: Endothelial cell behaviors in *Plgf*^{musc} transgenics.

(**A,B**) *In vivo* time-lapse imaging of endothelial cell nuclei distribution during aISV formation in WT (A) and in *plgf*^{musc} embryos (B). Individual endothelial cells are numbered. In WT initially three endothelial cells (indicated in yellow, numbered 1-3) migrated from the dorsal aorta into the prospective aISV territory; at this point endothelial cell number 2 divided and generated cell 2.1 (number in purple); resulting in an aISV consisting of four endothelial cells. In *plgf*^{musc} four endothelial cells migrated from the aorta (indicated in yellow, numbered 1-4); several proliferation events (cells derived from proliferation in purple) resulted in an aISV consisting of nine endothelial cells. EC nuclei distribution is representative for n=6 embryos analysed per group.

(**C,D**) *In vivo* confocal imaging of endothelial junctions in *Tg(fli1ep:gal4ff)*^{ubs4}*; Tg(UAS:VE-cadherinΔC-EGFP)*^{ubs12} transgenic WT (C) and *plgf*^{musc} embryo (D). Arrowheads delineate a single endothelial cell. Images represent n=33,38 aISVs per genotype examined over 2 separate experiments.

(**E,F**) *In vivo* confocal imaging of aISV endothelial nuclei distribution and vessel lumen in WT (E) and *plgf*^{musc} (F) using *Tg(fli1a:nEGFP)*^{y7}*; Tg(kdrl:hsa.HRAS-mCherry)*^{s916} double transgenic. Images represent n=29,32 aISVs per genotype derived from 2 separate experiments.

(**G**) *In vivo* time-lapse imaging of endothelial F-actin remodeling events in WT (upper panels) and *plgf*^{musc} (lower panels) using *Tg(fli1a:lifeactEGFP)*^{mu240}; and schematic representation of F-actin remodeling events, with F-actin in green. White arrowheads demarcate an individual endothelial cell. Illustrations are representative for each 4 examined biologically independent animals.

(**H-K**) *In vivo* confocal imaging of aISVs in WT treated with vehicle control (H), with Latrunculin-B (LatB) (I), with Rac1 inhibitor CAS 1177865-17-6 (J), with GEF Trio inhibitor ITX3 (K). Images are representative for n=29,17,38 and 44 aISVs per condition examined over 2 independent experiments.

(**L-O**) *In vivo* confocal imaging of aISVs in *plgf*^{musc} treated with vehicle control (L), with Latrunculin-B (LatB) (M), with Rac1 inhibitor CAS 1177865-17-6 (N), with GEF Trio inhibitor ITX3 (O). Images are representative for n=25 aISVs/condition examined over 3 independent experiments.

(**P**) Diameter quantification of images in H-K. Mean±s.e.m, unpaired two-sided students *t*-test. n=29aISVs from 8embryos (wt); 17aISVs from 6embryos (wt+Latb); 38aISVs from 10embryos (wt+Rac1-inh); 44aISV from 12embryos (wt+ITX3). #p<0.05 versus WT-DMSO.

Scale bar, 25 µm in all images. Hpf, hours post fertilization; EC, endothelial cell.

Supplementary Figure 7

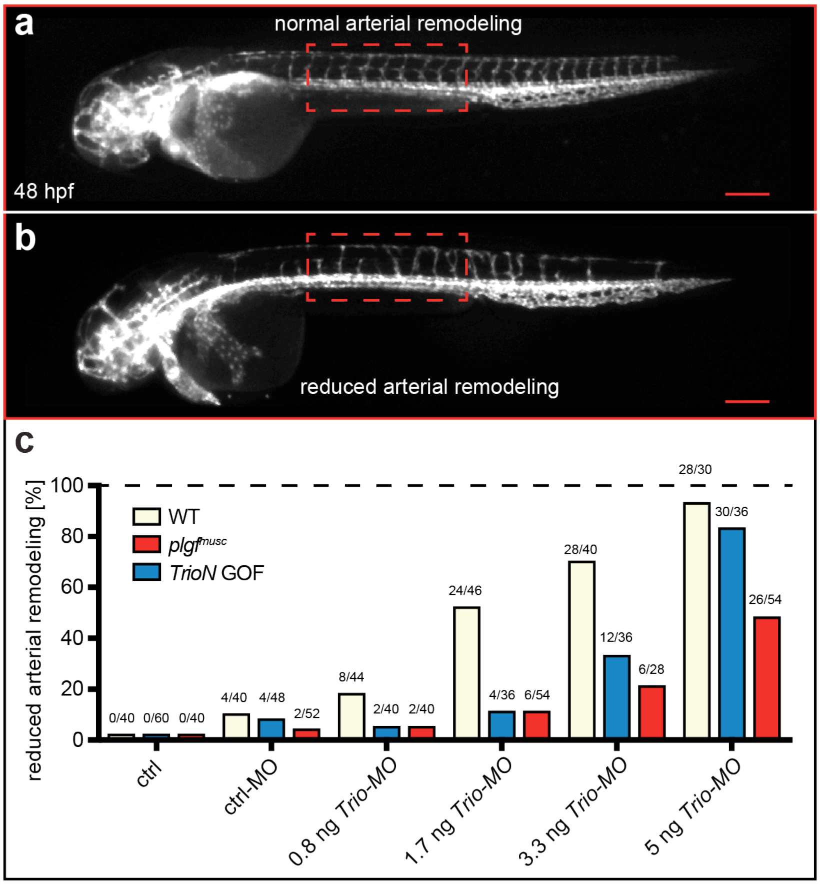

Supplementary Figure 7: Loss of Trio affects arterial remodeling.

(**A**) *In vivo* image of 48hpf embryo injected with control morpholino. Red dotted area indicates the area examined for analysing the arterial phenotype. n=120 animals examined over 2 separate experiments. Scale bar, 200µm.

(**B**) *In vivo* image of 48hpf embryo injected with 5ng Trio targeting morpholino. Red dotted area indicates the area examined for analysing the arterial phenotype. n=120 animals examined over 2 separate experiments. Scale bar, 200µm.

(**C**) Percentage of embryos showing reduced arterial remodeling in the trunk vasculature for indicated scenario. The percentage of embryos showing reduced arterial remodeling increased at higher dosages of Trio morpholino; overexpression of Plgf or TrioN reduced this percentage. Note that overexpression of Plgf provided a rescue from arterial remodeling deficits at all dosages investigated, in line with Plgf activating Trio *in vivo*. Precise numbers of animals with reduced arterial remodeling/ total animals analysed are indicated above the respective bar for each condition.

Supplementary Figure 8

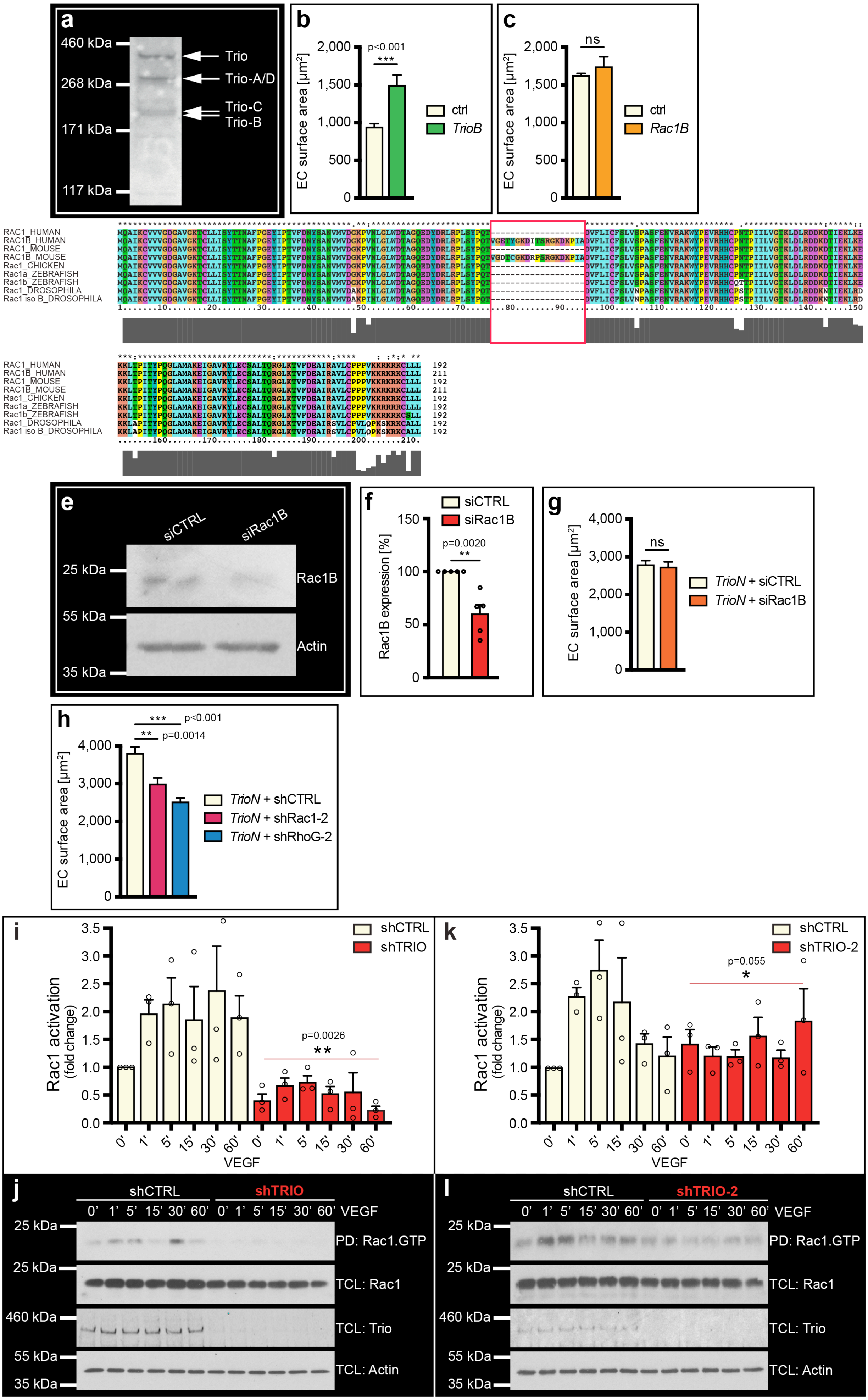

Supplementary Figure 8: Trio induced endothelial enlargement involves Rac1 and RhoG.

(**A**) Western Blot with an antibody directed against the spectrin domain (Antibody rbt CT232) of Trio using Vegf stimulated HUVEC lysate showing full-length TRIO (most upper band) and indicated TRIO splice-isoforms TRIO-A/D, TRIO-C and TRIO-B. Experiment was performed 3 times with reproducible result. Size marker (kDa) on the left. (**B**) Quantification of EC surface area after transfection with control plasmid, or with *TRIO-B*. Note the significant increase in EC size upon TRIO-B overexpression. Mean±s.e.m, unpaired two-sided students *t*-test, n=66,50 cells per indicated condition from 3 separate experiments. ***p<0.001.

(**C**) Quantification of EC surface area after transfection with control plasmid, or with *RAC1B*. Note no significant change upon RAC1B overexpression. 50 and 45 cells per indicated group were quantified over 3 independent experiments. Mean±s.e.m, unpaired two-sided students *t*-test. ns, not significant.

(**D**) Comparison of RAC1B protein sequence in different species. Multiple sequence analysis of human, mouse, chicken, drosophila and zebrafish Rac1. Aligned were the protein structures of human RAC1 with Rac1 in Mouse, Chicken, Zebrafish and Drosophila. Due to gene duplication zebrafish express two *rac1* co-orthologues: these genes are termed *rac1a* and *rac1b* – these are two different genes and not splice products. The zebrafish gene termed *rac1b*, should therefore not be confused with human *RAC1b*, which results from alternative splicing of the *RAC1* gene. The exon3 region encoding RAC1B in human and mouse is color indicated. Note that the RAC1B protein isoform contains an additional 19 amino acids (VGETYGRDITSRGKDKPIA, indicated by red box). This isoform was only observed in human and mouse, not in zebrafish, chicken or drosophila. Except for the exon3b region, a high level of protein homology was observed between Rac1 of the different species consistent with an evolutionary conserved functional role for Rac1.

(**E**) Western blot for RAC1B in endothelial cells upon control siRNA and siRAC1B transfection shows reduced RAC1B protein levels. Image represents results of 5 biologically independent experiments. Actin for protein loading control. Size marker (kDa) on the left.

(**F**) Quantification of RAC1B protein levels in endothelial cells transfected with control siRNA and siRAC1B examined over 5 biologically independent experiments. Mean±s.e.m, unpaired two-sided students *t*-test. **p=0.0020.

(**G**) Endothelial cells were silenced for *RAC1B* as indicated in (E) and subsequently transfected with *TrioN*. Loss of RAC1B had no impact on TrioN induced EC cell size increase. n=45 cells per group derived of 3 separate experiments. Mean±s.e.m, unpaired two-sided students *t*-test. ns, not significant.

(**H**) Changes in endothelial cell size upon *TrioN* gain-of-function after silencing of *Rac1* and *RhoG* using shRNA shRac1-2 and shRhoG-2 (as indicated). Mean±s.e.m, one-way ANOVA and posthoc dunnett's test, n=91,99,50 cells per indicated group examined over 3 separate experiments. Note: both loss of *Rac1* and *RhoG* reduced *TrioN* induced EC enlargement. **p=0.0014, ***p<0.001.

(**I**) Quantification of VEGF-A induced Rac1 activation in WT ECs (white bars, shCTRL), and after Trio silencing using shTrio-1 (red bars, shTRIO) – Western blot in (J). Mean±s.e.m, 2way-ANOVA, n=3 repeats per time-point and condition. **p=0.0026. The quantifications have always been corrected for the input.

(**J**) Western blot for active Rac1 (Rac1-GTP), total Rac1 (Rac1), upon VEGF; Trio for control of knockdown efficiency, and actin for protein loading control, as indicated. Experiment was performed 3 times with reproducible result. Size marker (kDa) on the left.

(**K**) Quantification of VEGF-A induced Rac1 activation in WT ECs (white bars, shCTRL), and after Trio silencing using shTrio-2 (red bars, shTRIO-2) – Western blot in (L). Mean±s.e.m, 2way-ANOVA, n=3 repeats per time-point and condition. *p=0.055. The quantifications have always been corrected for the input.

(**L**) Western blot for active Rac1 (Rac1-GTP), total Rac1 (Rac1), upon VEGF; Trio for control of knockdown efficiency, and actin for protein loading control, as indicated. Experiment was performed 3 times with reproducible result. Size marker (kDa) on the left.

Supplementary Figure 9

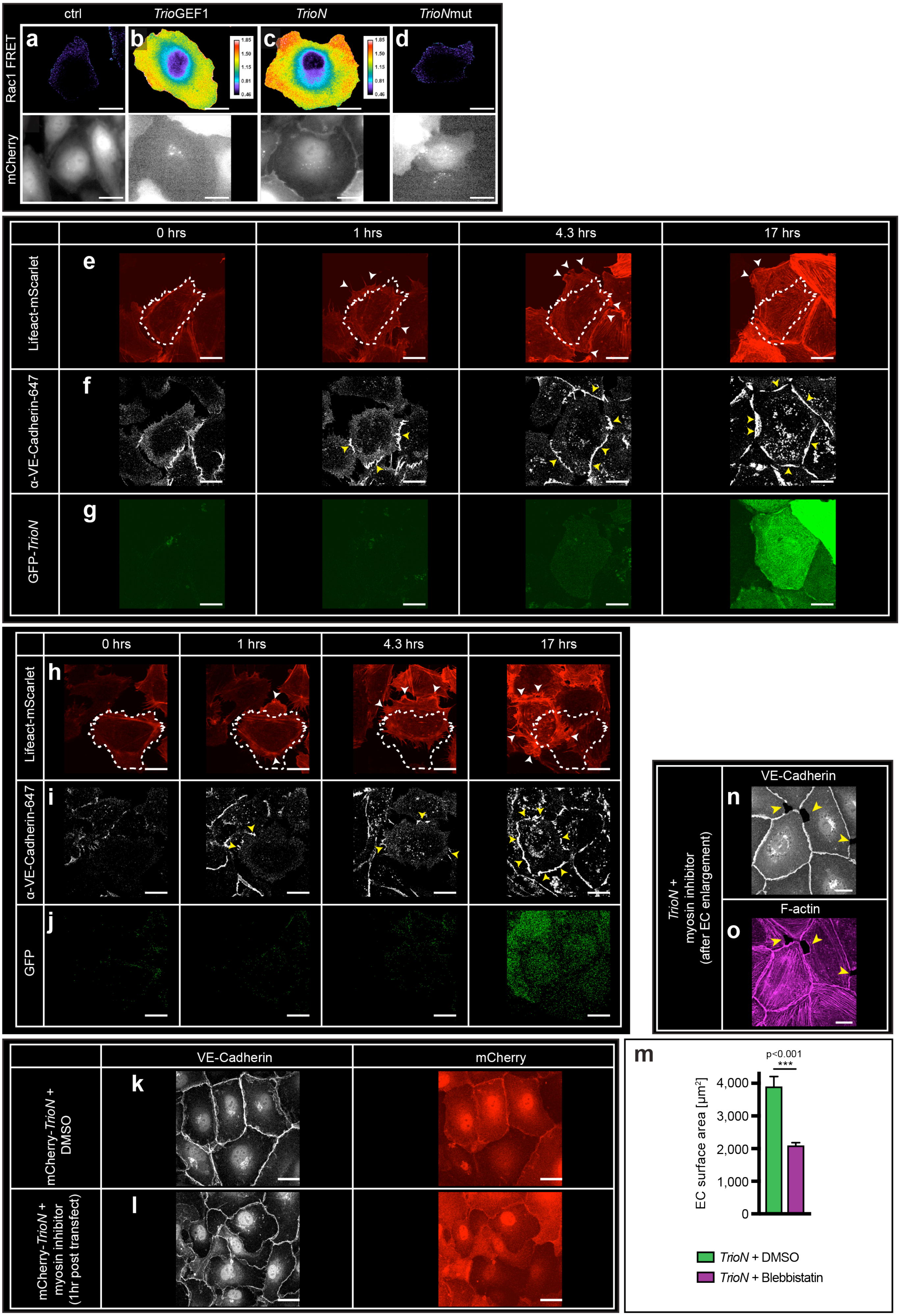

Supplementary Figure 9: Trio activates Rac1 and induces F-actin remodeling events in the cell-periphery.

(**A-D**) FRET ratio-signal (YFP/CFP) in confluent ECs upon transfection with *mCherry* only (Ctrl) (A); *mCherry-TrioGEF1* (*Trio*GEF1) (B); *mCherry-TrioN* (*TrioN*) (C); and *mCherry-TrioNmut* (*TrioN*mut) (D). Images are representative for n=90 cells per group of 3 independent experiments. FRET signals depicted as warm colors.

(**E**) Imaging of F-actin remodeling at indicated time points in endothelial cells transfected with *GFP-TrioN* and *lifeact-mScarlet*. Dotted line delineates endothelial cell boundary at start of imaging period. White arrowheads indicate lamellipodia extensions. Experiment was performed 5 times with reproducible result.
(**F**) Imaging of VE-cadherin based cell-cell junctions at indicated time points in endothelial cells transfected with *GFP-TrioN* and incubated with the non-blocking directly labeled anti-VE-Cadherin-647 antibody to visualize junctions (yellow arrowheads). Experiment was performed 5 times with reproducible result.
(**G**) Imaging of GFP expression in endothelial cells transfected with *GFP-TrioN* at indicated time points. Experiment was performed 5 times with reproducible result.

(**H**) Imaging of F-actin remodeling at indicated time points in endothelial cells transfected with *GFP-control* and *lifeact-mScarlet*. Dotted line delineates endothelial cell boundary at start of imaging period. White arrowheads indicate lamellipodia extensions. Experiment was performed 4 times with reproducible result.
(**I**) Imaging of VE-cadherin based cell-cell junctions at indicated time points in endothelial cells transfected with *GFP-control* and incubated with the non-blocking directly labeled anti-VE-Cadherin-647 antibody to visualize junctions (yellow arrowheads). Experiment was performed 4 times with reproducible result.
(**J**) Imaging of GFP expression in endothelial cells transfected with *GFP-control* at indicated time points. Experiment was performed 4 times with reproducible result.

(**K)** Imaging of VE-Cadherin in *mCherry-TrioN* transfected ECs treated with DMSO as control. Images represent n=140 cells examined over 3 independent experiments.
(**L**) Imaging of VE-Cadherin in *mCherry-TrioN* transfected ECs treated with the Myosin-II inhibitor Blebbistatin added at t=1h post transfection. Images represent n=130 cells examined over 3 independent experiments.

(**M**) Quantification of EC surface area upon Blebbistatin treatment; note significantly smaller cells. Mean±s.e.m, two-sided Mann Whitney U-test, n=64,43 cells per group. ***p<0.001.

(**N,O**) *TrioN* transfected ECs treated with MLCK inhibitor ML7 added at t=19h post transfection and imaging of VE-Cadherin (N), and F-actin (O). Note the emergence of “actin gaps” indicated by yellow arrowheads. Images represent n=150 cells examined over 4 independent experiments.

Scale bar, 25 µm in A-D, K, L, N, O; Scale bar, 20 µm in E-J. FRET, Fluorescence Resonance Energy Transfer.

Supplementary Figure 10

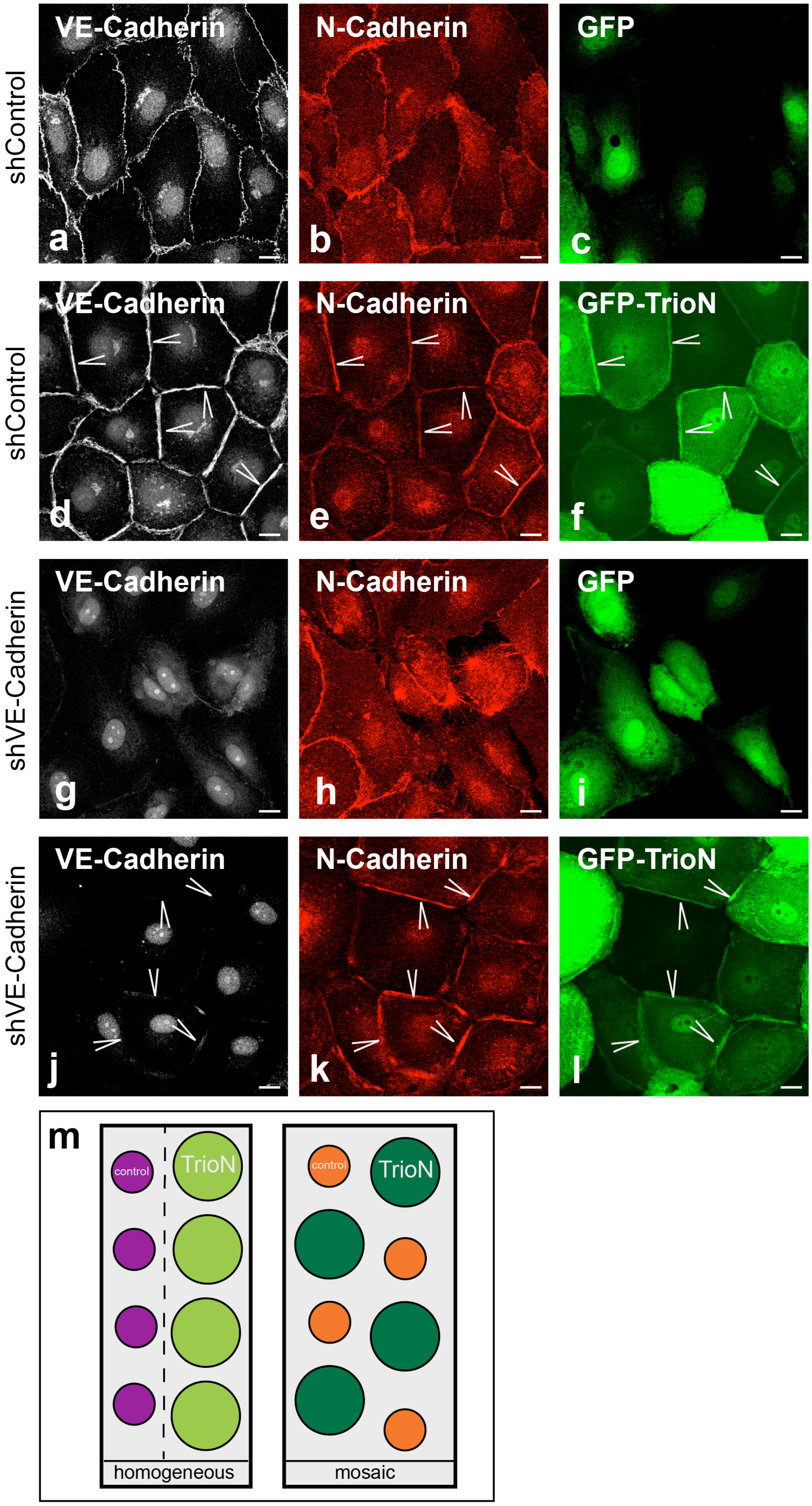

Supplementary Figure 10: N-Cadherin compensates for loss of VE-Cadherin in Trio gain of function endothelial cells.

(**A-C**) HUVECs were treated with shCTRL and transfected with *GFP* (C) and stained for VE-cadherin (A) and N-cadherin (B). Images represent n=180 analysed cells derived from 3 autonomous experiments. Scale bar, 20 µm.

(**D-F**) HUVECs were treated with shCTRL and transfected with *GFP-TrioN* (F) and stained for VE-cadherin (D) and N-cadherin (E). Arrowheads mark endothelial junctions. Images represent n=170 analysed cells derived from 3 autonomous experiments. Scale bar, 20 µm.

(**G-I**) HUVECs were treated with shVE-cadherin and transfected with *GFP* (I) and stained for VE-cadherin (G) and N-cadherin (H). Images represent n=180 analysed cells derived from 3 separate experiments. Scale bar, 20 µm.

(**J-L**) HUVECs were treated with shVE-cadherin and transfected with *GFP-TrioN* (L) and stained for VE-cadherin (J) and N-cadherin (K). Arrowheads mark endothelial junctions. Images represent n=180 analysed cells derived from 3 separate experiments. Scale bar, 20 µm.

(**M**) Measurement of EC cell size in a scenario in which control and *TrioN* expressing cells were not mixed – the homogeneous populations (left panel). In this scenario, *TrioN* expressing cells were facing neighbouring cells that expressed *TrioN*; whereas control transfected cells were facing control-transfected cells as neighbouring cells. This is indicated as non-mixed, homogenous populations. In the mosaic expression scenario *TrioN* cells were mixed with control plasmid expressing cells. EC size was measured in *TrioN* expressing cells that faced a neighbouring cell not expressing *TrioN* (mosaic expression).

Supplementary Figure 11

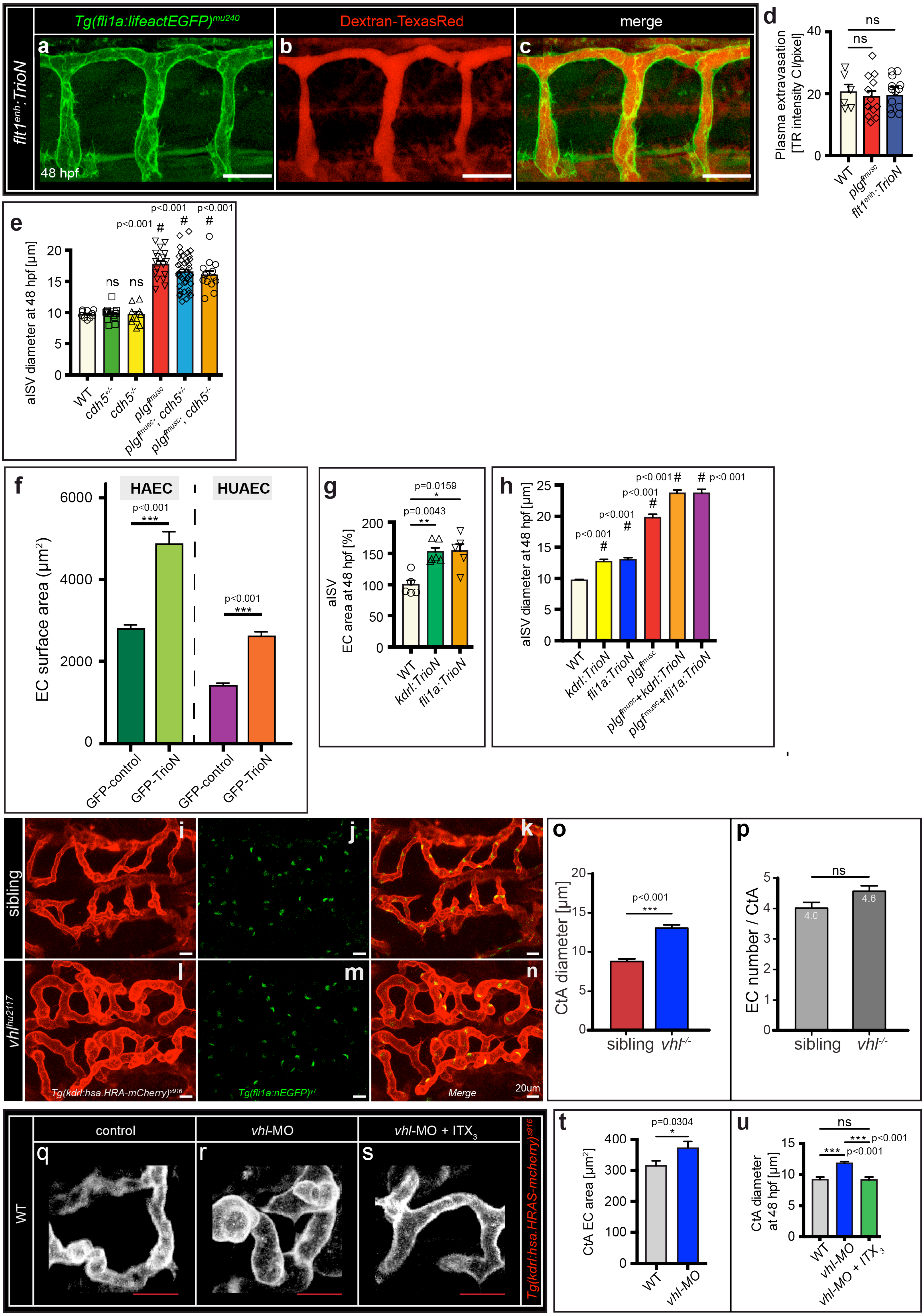

Supplementary Figure 11: Trio induced EC size increase response is conserved.

(**A-C**) *In vivo* imaging of plasma extravasation in *flt1^{enh}:TrioN* (n=17 embryos) injected with 70kD Dextran Texas-Red in 3 biologically independent experiments. Scale bar, 50µm.

(**D**) Quantification of plasma extravasation for indicated scenario. Mean±s.e.m; unpaired two-sided students *t*-test. n=6,13,14 embryos per group. ns, not significant.

(**E**) Quantification of aISV diameter in *ve-cadherin* (*cdh5^{ubs8}*) mutant. n=10,30,19,17,40 and 14 embryos per indicated genotype (each 4 aISVs/embryo) examined over 3 independent experiments. Mean±s.e.m; unpaired two-sided students *t*-test. # p<0.001 versus WT; ns, not significant.

(**F**) HAEC or HUAEC were transfected with *GFP-control* or *GFP-TrioN* construct, and cell area measured 24 hours upon transfection. Measured were n=116,68,329,130 cells per indicated group from 3 separate experiments, mean±s.e.m, unpaired two-sided students *t*-test, *** p<0.001, HAEC, Human aortic endothelial cells; HUAEC, Human umbilical artery endothelial cells.

(**G**) Quantification of EC area in aISV at 48hpf for indicated transgenic scenario. n=10,22,14 embryos/indicated group a 3 ECs/embryo derived from 3 separate experiments, mean±s.e.m, unpaired two-sided students *t*-test, * p=0.0159, ** p=0.0043.

(**H**) Quantification of aISV diameter at 48hpf for indicated transgenic scenario. n=24,28,21,18,21 and 18 aISVs per indicated condition examined over 3 autonomous experiments. Mean±s.e.m, unpaired two-sided students *t*-test, # p<0.001 versus WT.

(**I-N**) *In vivo* confocal imaging of cerebral vasculature (I,L) and endothelial cell nuclei (J,M), with merged image in K,N showing enlarged vessels in *vhl$^{-/-}$* mutants (*vhl^{hu2117}*) when compared with siblings. Images represent n=23,16 embryos per indicated genotype examined over 3 separate experiments. Scale bar, 20µm.

(**O,P**) Quantification of the images in K,N. Note the increase in diameter without significant change in EC numbers in *vhl$^{-/-}$*. n=86,95 CtAs/indicated genotype derived from 3 autonomous experiments. Mean±s.e.m, unpaired two-sided students *t*-test, *** p<0.001; ns, not significant.

(**Q-S**) *In vivo* confocal imaging of cerebral vasculature in WT (Q), *vhl* morphants (R), and *vhl* morphants treated with the Trio inhibitor ITX3 (S). Images represent n=21,23,20 embryos per condition examined over 3 independent experiments. Scale bar, 25µm.

(**T,U**) Quantification of CtA EC area (T), and CtA diameter (U) for indicated scenario. (T) n=21,17 ECs/indicated condition derived from 11,10 embryos per respective group examined over 3 separate experiments; (U) n=22,33,26 CtAs/group derived from 3 autonomous experiments. Mean±s.e.m, unpaired two-sided students *t*-test, *** p<0.001,* p=0.0304; ns, not significant.

Supplementary Figure 12

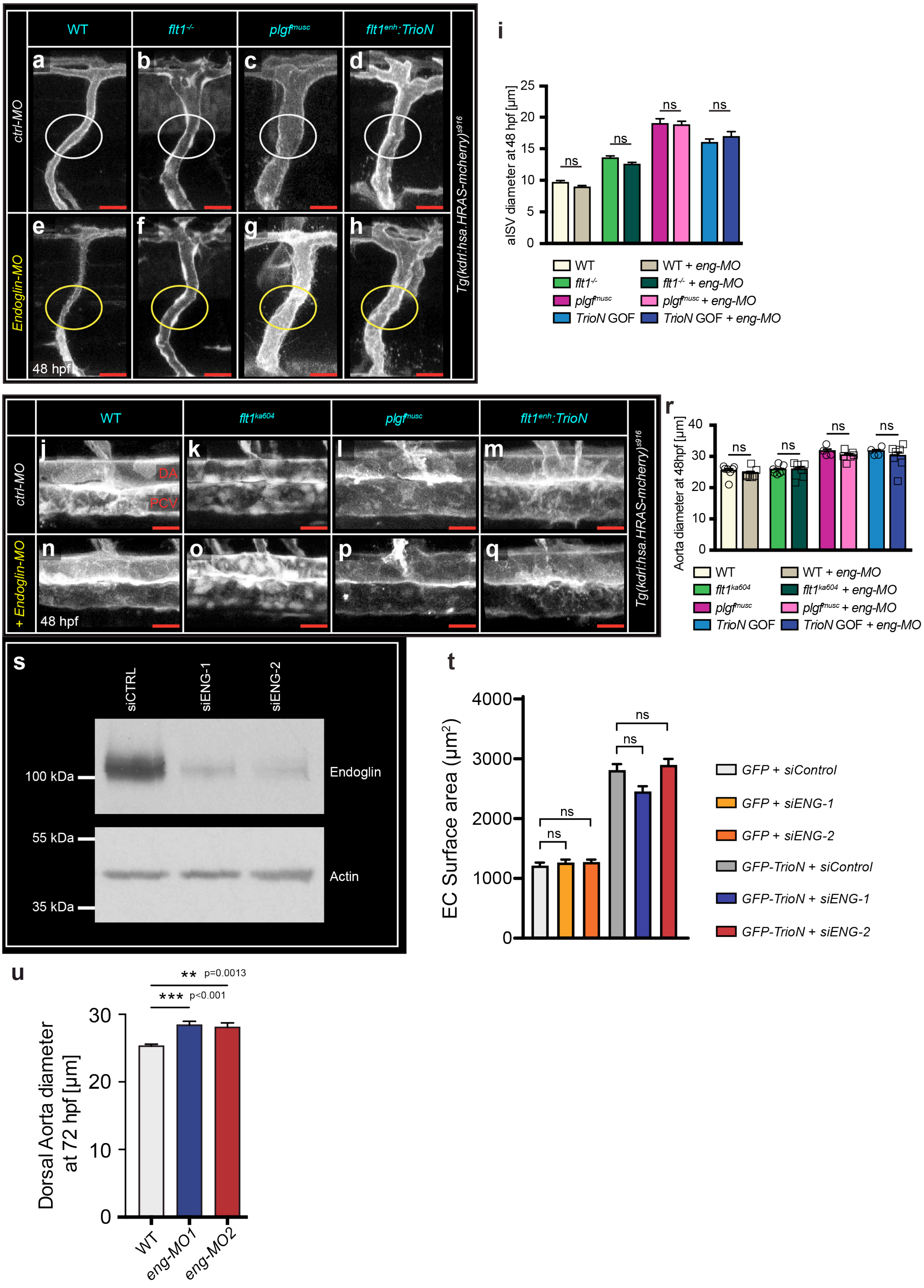

Supplementary Figure 12: Loss of *endoglin* and arterial size.

(**A-D**) *In vivo* confocal imaging of aISV in WT (A), *flt1*$^{-/-}$ (B), *plgf*^{musc} (C) and *flt1*^{enh}*:TrioN* (D). Images represent n=123,138,126,117 aISVs per indicated group derived from 4 independent experiments. Scale bar, 25µm.

(**E-H**) *In vivo* confocal imaging of aISV upon loss of *endoglin* in WT (E), *flt1*$^{-/-}$ (F), *plgf*^{musc} (G) and *flt1*^{enh}*:TrioN* (H). Images represent n=121,121,135,124 aISVs per indicated group examined over 4 independent experiments. Scale bar, 25µm.

(**I**) Quantification of images in A-H. n=20,24,17,23,18,22,21 and 23 aISVs per indicated scenario derived from 4 biologically independent experiments. Mean±s.e.m, unpaired two-sided students *t*-test; ns, not significant.

(**J-M**) *In vivo* confocal imaging of the dorsal aorta at 48hpf for indicated genotype. Images are representative for n= 28,35,34,39 embryos/group examined over 3 independent experiments. Scale bar, 25µm.

(**N-Q**) *In vivo* confocal imaging of the dorsal aorta at 48hpf for indicated genotype after morpholino mediated knock-down of *endoglin*. Images are representative for n= 34,34,38,35 embryos/group examined over 3 independent experiments. Scale bar, 25µm.

(**R**) Quantification of the images in J-Q. n=7,7,7,6,4,6,4 and 7 embryos per indicated scenario derived from 3 biologically independent experiments. Mean±s.e.m, unpaired two-sided students *t*-test; ns, not significant. Note that morpholino mediated loss *of endoglin* had no impact on aorta size at this stage of development.

(**S**) Western blot *Endoglin* (ENG) for control of knockdown efficiency using two different siRNA smart pools siENG-1 and siENG-2, and actin for protein loading control, as indicated. Image represents results of 3 biologically independent experiments. Size marker (kDa) on the left.

(**T**) Changes in endothelial cell size upon *TrioN* gain of function after silencing of *Endoglin* using two different siRNA smart pools siENG-1 and siENG-2 (as indicated); n=110 cells/indicated group examined over 2 biologically independent experiments. Mean±s.e.m, Kruskal-Wallis-test, ns, not significant. Note: *TrioN* induced EC enlargement is independent of *Endoglin*.

(**U**) Quantification of aorta diameter at 72hpf in *endoglin* morphants. *Endoglin* was targeted using two different morpholinos (MO1 & MO2). Note the significant increase in aorta diameter in line with the aorta diameter changes reported in *endoglin* mutants at 72hpf. n=11,14,14 embryos/indicated group examined over 2 separate experiments; mean±s.e.m, unpaired two-sided students *t*-test; *** p<0.001; ** p=0.0013.

Supplementary Figure 13

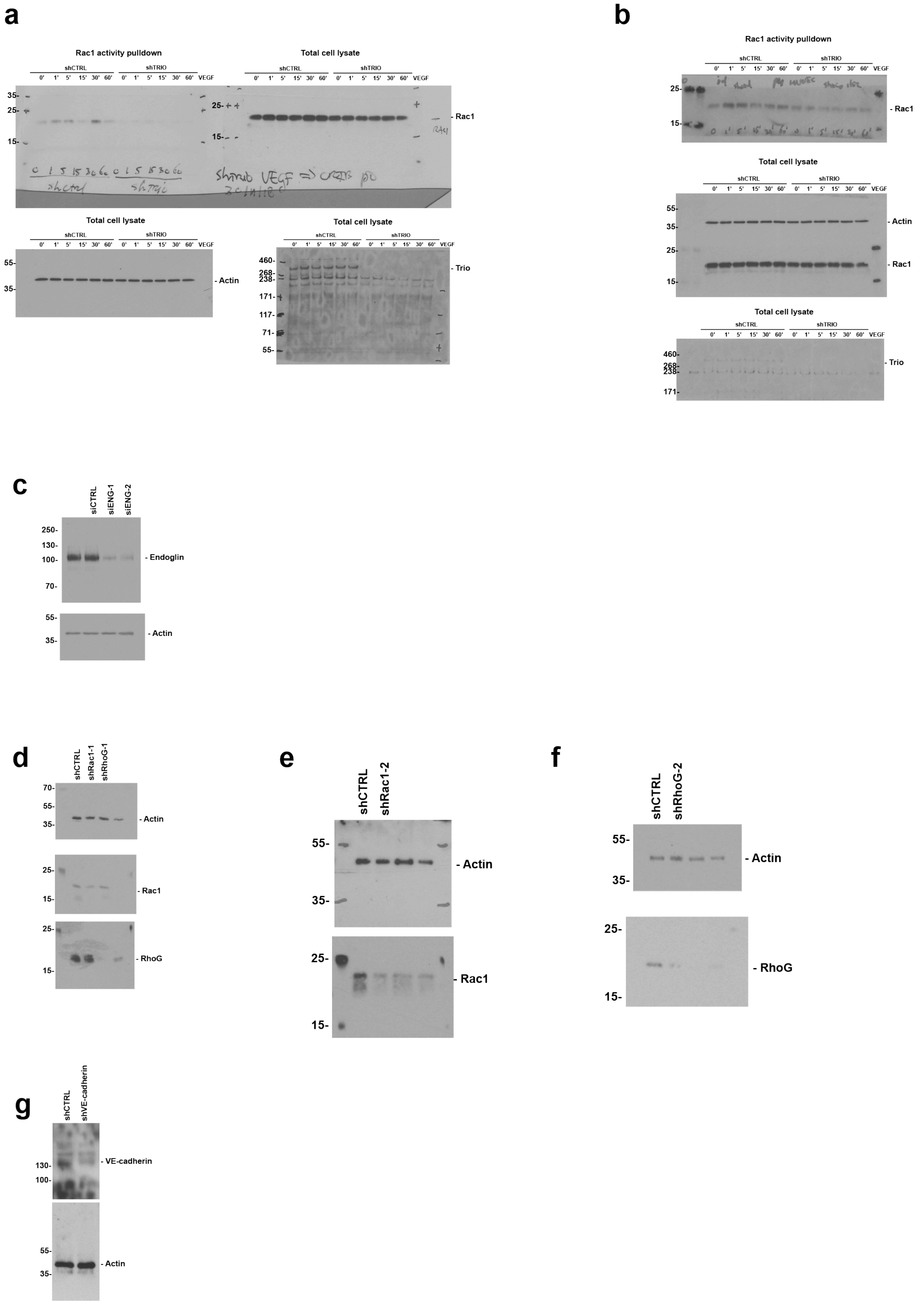

Supplementary Figure 13: Full Western blots of indicated scenarios

(**A**) Rac1 activity pulldown after VEGF treatment in HUVECs 72 hours after transduction with a control non-targeting shRNA or shRNA construct targeting Trio (shTrio). Protein extracts were probed with antibodies directed against Rac1 (21 kDa), Trio (350 kDa) and actin (42 kDa). Size marker (kDa) on the left.

(**B**) Rac1 activity pulldown after VEGF treatment in HUVECs 72 hours after transduction with a control non-targeting shRNA or a second shRNA construct targeting Trio (shTrio-2). Protein extracts were probed with antibodies directed against Rac1 (21 kDa), Trio (350 kDa) and actin (42 kDa). Size marker (kDa) on the left.

(**C**) Immunoblot analysis of protein extracts prepared from HUVECs 72 hours after transduction with a control non-targeting shRNA or two siRNA constructs targeting Endoglin (siENG-1 and siENG-2). Extracts were probed with antibodies directed against Endoglin (95 kDa) and actin (42 kDa). Size marker (kDa) on the left.

(**D**) Immunoblot analysis of protein extracts prepared from HUVECs 72 hours after transduction with a control non-targeting shRNA or shRNA constructs targeting Rac1 or RhoG (shRac1-1 and shRhoG-1). Extracts were probed with antibodies directed against Rac1 (21 kDa), RhoG (20 kDa) and actin (42 kDa). Size marker (kDa) on the left.

(**E**) Immunoblot analysis of protein extracts prepared from HUVECs 72 hours after transduction with a control non-targeting shRNA or a second shRNA construct targeting Rac1 (shRac1-2). Extracts were probed with antibodies directed against Rac1 (21 kDa) and actin (42 kDa). Size marker (kDa) on the left.

(**F**) Immunoblot analysis of protein extracts prepared from HUVECs 72 hours after transduction with a control non-targeting shRNA or a second shRNA construct targeting RhoG (shRhoG-2). Extracts were probed with antibodies directed against RhoG (20 kDa) and actin (42 kDa). Size marker (kDa) on the left.

(**G**) Immunoblot analysis for VE-Cadherin protein upon transfection with control shRNA and shRNA targeting *VE-Cadherin*, with loading control actin. Size marker (kDa) on the left.

Supplementary Table 1. Expression of RAC1 and RAC1B in human endothelial cells of different origins

Transcript ID	BECs	HAoEC	HHSEC	HMVEC-D	HUVEC
ENST00000348035	131.345±9.35	82.970±36.61	33.578±0.00	155.024±0.00	89.816±38.24
ENST00000356142	Not detected	Not detected	Not detected	Not detected	35.099±12.25

ENST00000348035 encodes for *RAC1*
ENST00000356142 encodes for *RAC1B*

(BEC, blood vascular endothelial cell; HAoEC, human aortic endothelial cell; HHSEC, human hepatic sinusoidal endothelial cell; HMVEC, human lung microvascular endothelial cell; HUVEC, human umbilical vein endothelial cell).

Comparison of RAC1 and RAC1B expression levels in human endothelial cells derived from 5 different regions using public available RNA deep sequencing databases (database: https://doi.org/10.1038/srep32475)[1]. Note that only HUVEC expressed both Rac1 and Rac1b.

Supplementary Table 2. Oligonucleotide sequences

Oligonucleotide name	Oligonucleotide sequence
sFlt1_HAHA_ODN_1	5'-CTTATTCTTTATAGCGGCCCGGCGCGGCTGGCCTACCCATACGACGTCCCAGACTACGCTTACCCATACGACGTCCCAGACTACGCTTGAGGTGGAGGAGGCCCAGGGAGAGTGGAGTTGTGG-3'
flt1_E3_gDNA_fw	5'-CAGCTCAACACACACAGTATTGTTTTA-3'
flt1_E3_gDNA_rev	5'-ACACCTGAAGCATCTTACCTGTGA-3'
SDM_Flt1delta7_fw	5'-ATGAAGGAGGGAGAGCCCTT-3'
SDM_Flt1delta7_rev	5'-CGACTGCACCTTCACAAAAGG-3'
sflt1_fw	5'-ACTATCCTTACTGGGATCCAGC-3'
HA_rev	5'-TGGGACGTCGTATGGGTAAGCG-3'
SmaI_Plgf_CDS_fw	5'-ATCCCCGGGAGTTATTTGACGTCACTGTTACG-3'
XhoI_Plgf_CDS_rev	5'-ATCCTCGAGTTACCTGCGGGGAGGCTGACACCGG-3'
SmaI_Vegfb_CDS_fw	5'-ATC CCC GGG GGGATCAAGCTGAGGGTTA-3'
XhoI_Vegfb_CDS_rev	5'-AGT CTC GAG CTATTCACTTTGGGTTTCACATC-3'
Flt1_XhoI_fw	5'-GAAGTCTCGAGACCATGGGTTTCGATATATTATTTG-3'
sFlt1_HAHA_XbaI	5'-AGGTCTCTAGATTAAGCGTAGTCTGGGACGTCGTATGGGTAAGCGTAGTCTGGGACGTCGTATGGGTATGCGAGTCTAGCCGGGCC-3'

Supplementary Table 3. Inhibitory shRNAs

shRNA name	shRNA identity	Source
shTRIO	(TRC_297115)	Sigma mission library
shTRIO-2	(TRC_196250)	Sigma mission library
shRac1-1	(TRC_4872)	Sigma mission library
shRac1-2	(TRC_4873)	Sigma mission library
shRhoG-1	(TRC_48019)	Sigma mission library
shRhoG-2	(TRC_48018)	Sigma mission library
shVE-Cadherin	(TRC_54090)	Sigma mission library

Supplementary Table 4. Inhibitory siRNAs

siRNA name	siRNA identity	siRNA sequence	Source
siENG-1	(s4677)	5'-UGACCUGUCUGGUUGCACAtt-3'	Ambion pre-designed siRNA
siENG-2	(s4678)	5'-GGACUGUCUUCAUGCGCUUtt-3'	Ambion pre-designed siRNA
siRac1B	-	5'-AAACGUACGGUAAGGAUAUAACCTC-3' / 3'-UCUUUGCAUGCCAUUCCUAUAUUGGAG-5'	Custom designed

Supplementary Table 5. Inhibitors

Target	Inhibitor	Final concentration	Dissolved in	Manufacturer
Kdrl/ VEGFR2	ki8751	0.125 µM (low)<br>0.25 µM (high)	DMSO	Sigma, St Louis, MO, USA
F-actin polymerisation	Latrunculin B	0.15 µg/ml	DMSO	Sigma, St Louis, MO, USA
Rac1	CAS 1177865-17-6 (synonyme: NSC23766)	100 µM	DMSO	Calbiochem, Merck KGaA, Germany
TrioN	ITX3	100 µM<br>200 µM	DMSO	Sigma, St Louis, MO, USA
Non-muscle myosin II	(±)-Blebbistatin (CAS 674289-55-5)	50 µM	DMSO	Sigma, St Louis, MO, USA
MLCK	ML7	5 µM	DMSO	Sigma, St Louis, MO, USA

Supplementary Table 6. Chemicals

Chemical	Final concentration	Dissolved in	Manufacturer
(E/Z)-Endoxifen Hydrochloride Hydrate	0.5 µM	E3 medium	Sigma, St Louis, MO, USA
Dextran, Texas Red™ 70 000 MW	2 mg/ml	E3 medium	Thermo Fisher Scientific
Nifedipine	15 µM	DMSO	Sigma, St Louis, MO, USA

Supplementary Table 7. Antibodies and dyes

1° Antibody	Manufacturer; Cat. No.	Dilution or concentration	2° Antibody	Manufacturer; Cat. No.	Dilution
anti-alpha-tubulin (clone DM1A)	Sigma; T6199	for WB: 1:5000	donkey anti-goat IgG (H+L) DyLight-405	Jackson ImmunoResearch; 705-475-147	for IF: 1:200

anti-beta-actin (clone AC-40)	Sigma; A3853	for WB: 1:1000	donkey anti-mouse IgG (H+L) Alexa Fluor 568	Thermo Fisher Scientific; A-10037	for IF: 1:200
anti-ENG (3A9)	Cell Signaling; 14606	for WB: 1:1000	goat anti-mouse-HRP	Dako; P0447	for WB: 1:7000
anti-HA (HA.11)	Covance; MMS-101P	for IHC: 1:1000	goat anti-mouse IgG (H+L) Alexa Fluor 488	Invitrogen; A-11029	for IHC: 1:1,000
anti-integrin alpha5 (clone EPR784)	Abcam; ab150361	for IF: 1:100	rabbit anti-goat-HRP	Dako; P0449	for WB: 1:7000
anti-integrin beta1 (clone P5D2)	Abcam; ab24693	for IF: 1:100	swine anti-rabbit-HRP	Dako; P0399	for WB: 1:7000
anti-N-Cadherin (3B9)	Zymed; 180224	for IF: 1:100			
anti-phospho-Paxillin (pTyr118)	Thermo Fisher Scientific; 44722G	for IF: 1:100			
anti-pMLC-S19	Cell Signaling; 3675	for IF: 1:100			
anti-pMLC-T18S19	Cell Signaling; 3674	for IF: 1:100			
anti-Rac1 (clone 102)	BD Biosciences; 610651	for WB: 1:1000			
anti-Rac1B	MERCK; 09-271	for WB: 1:500			
anti-RhoG [1F3 B3 E5]	Santacruz; sc-80015	for WB: 1:250			
anti-spectrin (clone CT232)	kind gift from Dr. Betty Eipper (UConne Health, Farmington, USA); doi: 10.1016/j.gene.2004.12.028	for WB: 1:1000			
anti-Tiam1 [C-16]	Santacruz; sc-872	for IF: 1:100			
anti-Trio (clone D-20)	Santacruz; sc-6060	for WB: 1:100			
anti-Vav2 [H-200]	Santacruz; sc-20803	for IF: 1:100			
anti-VE-		for IF:			

Cadherin (clone C-19)	Santacruz; sc-6458	1:100 for WB: 1:1000			
anti-VE-Cadherin-Alexa Fluor 647 (clone 55-7H1)	BD Biosciences; 561567	for live imaging: 1:200			
anti-VEGFR2 (55B11)	Cell Signaling; 2479	for IF: 1:100			
**Staining dye**	**Manufacturer**	**Dilution or concentration**			
phalloidin promofluor-405	Promocell; PK-PF405-7-01	for IF: 1:100			

Supplementary References

1. Müller, R. *et al.* ANGIOGENES: Knowledge database for protein-coding and noncoding RNA genes in endothelial cells. *Sci. Rep.* **6**, (2016).
