# Supplementary material for: The GEF Trio controls endothelial cell size and arterial remodeling downstream of Vegf signaling in both zebrafish and cell models
Source: Nat Commun. 2020 Oct 21;11:5319. doi: 10.1038/s41467-020-19008-0 (PMC7578835; doi:10.1038/s41467-020-19008-0)
Supplement: Supplementary file 11 — Description of Additional Supplementary Files [file 41467_2020_19008_MOESM11_ESM.pdf]

Title: Supplementary Movie 1: *In vivo* imaging of trunk perfusion in WT embryo.
Description: Imaging of red blood cell perfusion of trunk aISV and vISV in WT embryo at 2dpf. Anterior to the left, dorsal is up. 25 frames/sec.

Title: Supplementary Movie 2: *In vivo* imaging of trunk perfusion in *plgf^{musc}* embryo.
Description: Imaging of red blood cell perfusion of trunk aISV and vISV in *plgf^{musc}* embryo at 2dpf. Anterior to the right, dorsal is up. 25 frames/sec.

Title: Supplementary Movie 3: *In vivo* imaging of aISV perfusion in *plgf^{musc}* embryo.
Description: Imaging of red blood cell perfusion in aISV of *plgf^{musc};Tg(kdrl:hsa.HRAS-mcherry)^{s916}* transgenic embryo at 2dpf. RBCs in transmitted light, endothelial cell membranes in red. Scale bar represents 10 µm. aISV, arterial intersegmental vessel. Note that the aISV lumen is enlarged thereby allowing multiple erythrocytes to pass at the same time. Dorsal is up.

Title: Supplementary Movie 4: *In vivo* imaging of aISV growth in WT embryo.
Description: Time-lapse confocal imaging of a growing aISV in *Tg(fli1a:lifeactEGFP)^{mu240}* transgenic line. Developmental stage and scale bar are indicated (bottom left and right corner, respectively). Scale bar represents 20 µm. aISV, arterial intersegmental vessel.

Title: Supplementary Movie 5*: In vivo* imaging of aISV growth in *plgf^{musc}* embryo.
Description: Time-lapse confocal imaging of a growing aISV in the *plgf^{musc}*; *Tg(fli1a:lifeactEGFP)^{mu240}* double transgenic line. Developmental stage and scale bar are indicated (bottom left and right corner, respectively). Scale bar represents 20 µm. aISV, arterial intersegmental vessel.

Title: Supplementary Movie 6: Endothelial shape change upon transfection with *TrioN.*
Description: Time-lapse confocal imaging of endothelial cell shape changes in cells transfected with *Tri oN-GFP* (green) and Lifeact-mScarlet for visualizing F-actin remodeling (in red). Cells were imaged for 17 hours (30 frames/hour). VE-Cadherin (in white) was visualized using a non-blocking alexa-647-labelled VE-Cadherin antibody. Movie framerate, 20 frames/second. Scale bar represents 20 µm. EC, endothelial cell.

Title: Supplementary Movie 7: Endothelial shape change upon control transfection.
Description: Time-lapse confocal imaging of endothelial cell shape changes in cells transfected wi th control GFP construct (green), and Lifeact-mScarlet for visualizing F-actin remodeling (in red). Cells were imaged for 17 hours (30 frames/hour). VE-Cadherin (in white) was visualized using a non-blocking alexa-647-labelled VE-Cadherin antibody. Movie framerate, 20 frames/second. Scale bar represents 20 µm. EC, endothelial cell.

Title: Supplementary Movie 8: *In vivo* imaging of trunk perfusion in *plgf^{musc}*; *flt1^{enh}:TrioN* embryo.
Description: Imaging of red blood cell perfusion of trunk aISV and vISV in *plgf^{musc}* ; *flt1^{enh}:TrioN* embryo at 2dpf. Anterior to the right, dorsal is up. 25 frames/sec.
