# Supplementary material for: The GEF Trio controls endothelial cell size and arterial remodeling downstream of Vegf signaling in both zebrafish and cell models
Source: Nat Commun. 2020 Oct 21;11:5319. doi: 10.1038/s41467-020-19008-0 (PMC7578835; doi:10.1038/s41467-020-19008-0)
Supplement: Supplementary file 12 — Reporting Summary [file 41467_2020_19008_MOESM12_ESM.pdf]

nature research

Corresponding author(s): Ferdinand le Noble

Last updated by author(s): Aug 19, 2020

Reporting Summary

Nature Research wishes to improve the reproducibility of the work that we publish. This form provides structure for consistency and transparency in reporting. For further information on Nature Research policies, see our Editorial Policies and the Editorial Policy Checklist.

Statistics

For all statistical analyses, confirm that the following items are present in the figure legend, table legend, main text, or Methods section.

n/a	Confirmed	
☐	☒	The exact sample size (n) for each experimental group/condition, given as a discrete number and unit of measurement
☐	☒	A statement on whether measurements were taken from distinct samples or whether the same sample was measured repeatedly
☐	☒	The statistical test(s) used AND whether they are one- or two-sided<br>*Only common tests should be described solely by name; describe more complex techniques in the Methods section.*
☒	☐	A description of all covariates tested
☐	☒	A description of any assumptions or corrections, such as tests of normality and adjustment for multiple comparisons
☐	☒	A full description of the statistical parameters including central tendency (e.g. means) or other basic estimates (e.g. regression coefficient) AND variation (e.g. standard deviation) or associated estimates of uncertainty (e.g. confidence intervals)
☒	☐	For null hypothesis testing, the test statistic (e.g. F, t, r) with confidence intervals, effect sizes, degrees of freedom and P value noted<br>*Give P values as exact values whenever suitable.*
☒	☐	For Bayesian analysis, information on the choice of priors and Markov chain Monte Carlo settings
☒	☐	For hierarchical and complex designs, identification of the appropriate level for tests and full reporting of outcomes
☒	☐	Estimates of effect sizes (e.g. Cohen's d, Pearson's r), indicating how they were calculated

Our web collection on statistics for biologists contains articles on many of the points above.

Software and code

Policy information about availability of computer code

Data collection	Leica Las X 3.5.6.21594, Zeiss Zen 2 software
Data analysis	Adobe Illustrator CS6 & CC 2020, Adobe Photoshop CS6 & 2020, Fiji [(Fiji Is Just) ImageJ 2.0.0-rc-69/1.52p & 1.52i], GraphPad Prism 6.0 & 8.0, Microsoft Excel 16.16.2, Cell Profiler 3.1.9, Matlab 2012a

For manuscripts utilizing custom algorithms or software that are central to the research but not yet described in published literature, software must be made available to editors and reviewers. We strongly encourage code deposition in a community repository (e.g. GitHub). See the Nature Research guidelines for submitting code & software for further information.

Data

Policy information about availability of data

All manuscripts must include a data availability statement. This statement should provide the following information, where applicable:

- Accession codes, unique identifiers, or web links for publicly available datasets
- A list of figures that have associated raw data
- A description of any restrictions on data availability

The authors declare that all data supporting the findings of this study are available within the article and its Suplementary files or from the corresponding author upon reasonable request. Original raw data blots for cropped images in Figure 4n, Supplementary Figure 8a,e,j,l and Supplementary Figure 12s are provided in Supplementary Figure 14.

Field-specific reporting

Please select the one below that is the best fit for your research. If you are not sure, read the appropriate sections before making your selection.

- [x] Life sciences
- [ ] Behavioural & social sciences
- [ ] Ecological, evolutionary & environmental sciences

For a reference copy of the document with all sections, see nature.com/documents/nr-reporting-summary-flat.pdf

Life sciences study design

All studies must disclose on these points even when the disclosure is negative.

Sample size

Sample sizes were not statistically predetermined but were chosen based on previous experience, standards in the fields and previously published literature.

Data exclusions

No data were excluded.

Replication

All images shown in the figures are representative examples of the respective phenotypes and expression patterns. The following section indicates how often experiments have been repeated independently with similar results. Fig. 1a,b, representative micrographs from n=48, and n=32 biologically independent embryos per indicated condition. Fig. 1c,d, images are representative for n=24 embryos per condition examined over 3 independent experiments. Fig. 1h-k, n=112,86,126,93 aISVs per genotype derived from 4 autonomous experiments. Fig. 1l, mean±s.e.m, unpaired two-sided students t-test, n=18,16,16,10 aISVs per genotype. ***p<0.001. Fig. 1m, n=56 aISVs examined over 3 independent experiments. Fig. 1n, mean±s.e.m, unpaired two-sided students t-test, n=20,25 aISVs for indicated scenario, ***p<0.001. Fig. 1o,p, plasma extravasation in WT (n=14) and plgfmusc (n=16 embryos) injected from 3 biologically independent experiments. Fig. 1q, mean ±s.e.m; unpaired two-sided students t-test, n=6,8 embryos per indicated group. ns, not significantly different.

Fig. 2d, mean±s.e.m, unpaired two-sided students t-test, n=7,13 and 24 aISVs for indicated scenario. **p=0.0051, ***p<0.001. Fig. 2e-g, representative image of indicated scenario (n=40, 19, 13 respectively). Fig. 2h, mean±s.e.m, unpaired two-sided students t-test, n=40,19 and 13 aISVs for indicated scenario, from 3 independent experiments. ns, not significant. **p<0.01.

Fig. 3a-d, representative micrographs from 3 biologically independent experiments. Fig. 3e, mean±s.e.m, one-way ANOVA & posthoc bonferroni test, n=18,20,20,15,20,20 aISVs for indicated scenarios. **p=0.0026, ***p<0.001. Fig. 3f, mean±s.e.m, one-way ANOVA & posthoc bonferroni test, n=15,22,14,28 aISVs for indicated scenarios. ***p<0.001. Fig. 3k, n=21,16,42,34 aISVs per indicated genotype, mean±s.e.m, unpaired two-sided students t-test. ns, not significant, ***p≤0.001. Fig. 3l,m, representative micrographs from 83 and 94 images, from 3 biologically independent experiments. Fig. 3n, mean±s.e.m, unpaired two-sided students t-test, n=16 aISVs/group. ***p<0.001. Fig. 3 o-q, representative micrographs from n=48,55,57 images per condition, from 3 biologically independent experiments. Fig. 3r, mean±s.e.m, unpaired two-sided students t-test, n=11,20 and 15 aISVs per treatment group. ***p<0.001. Fig. 3v, mean±s.e.m, unpaired two-sided students t-test, n=20 aISVs per group. ***p<0.001.

Fig. 4a,b, n=6 biologically independent animals per group. Fig. 4c, mean±s.e.m, unpaired two-sided students t-test, n=10,11 aISVs for indicated genotype. *p=0.0190, ***p<0.001. Fig. 4d, illustrations are representative for each 4 examined biologically independent animals. Fig. 4e, mean±s.e.m, unpaired two-sided students t-test, n=6 ECs for each genotype. Surface area expansion velocity is indicated as average slope (in µm2 h-1) calculated from regression analysis. Fig. 4f, mean±s.e.m, one-way ANOVA & posthoc bonferroni test, n=25,38,30,60,59 aISVs for indicated condition. ***p<0.001. Fig. 4g-i, n=67,59,60 aISVs examined over 3 autonomous experiments. Fig. 4j-l, n=53,48,62 micrographs from 3 independent experiments. Fig. 4m, mean±s.e.m, one-way ANOVA & posthoc bonferroni test, n=17,21,18,15,17 and 16 aISVs/group. ***p<0.001. Fig. 4n-p, n=58,58,59 aISVs examined over 3 independent experiments. Fig. 4q-s, n=66,58,59 aISV micrographs/ condition from 3 independent experiments. Fig. 4t, mean±s.e.m, one-way ANOVA & posthoc bonferroni test, n=18,14,14,16,18 and 18 aISVs/ group. ns, not significant, **p<0.01, ***p<0.001.

Fig. 5a-f, images are representative for n=120 ECs/group derived from 3 biologically independent experiments. Fig. 5g, mean±s.e.m, unpaired two-sided students t-test, n=59,69,63 cells per indicated group respectively. ***p<0.001. Fig. 5h-k, images are representative for n=130 ECs per indicated group derived from 3 biologically independent experiments. Fig. 5l, mean±s.e.m, two-sided Mann Whitney U-test, n=67,56,65,74 cells per indicated group respectively. *p=0.0477; ***p<0.001. Fig. 5m, mean ± s.e.m, unpaired two-sided students t-test, n=89,83 cells per indicated group respectively, derived from 3 independent experiments. ***p<0.001. Fig. 5o, mean ± s.e.m, unpaired two-sided students t-test, n=73,97 cells per indicated group, examined over 3 independent experiments. ***p<0.001. Fig. 5p, mean ± s.e.m, unpaired two-sided students t-test, n=101,146 cells per indicated group examined over 3 independent experiments. ***p<0.001. Fig. 5q-s, images are representative for n=120 ECs per group derived from 3 biologically independent experiments. Fig. 5t-v, images are representative for n=120 ECs per group derived from 3 biologically independent experiments. Fig. 5w, cell size was measured of n=78,82,103 and 80 cells for indicated scenario respectively, derived from 3 separate experiments. Mean ± s.e.m, unpaired two-sided students t-test. ns, not significant, ***p<0.001.

Fig. 6a, images are representative for n=100 cells per group of 3 independent experiments. Fig. 6b, images are representative for n=115 cells per group of 3 independent experiments. Fig. 6c,d, mean±s.e.m, two-sided Mann Whitney U-test, n=69,59,65,69 cells per indicated group (c). mean±s.e.m, two-sided Mann Whitney U-test, n=69,59,65 and 69 cells per group (d). ***p<0.001. Fig. 6e, images are representative for n=60 cells per condition derived from 3 independent experiments. Fig. 6f, (left panel) mean±s.e.m, unpaired two-sided students t-test, n=50,110 cells per group. (right panel) mean±s.e.m, unpaired two-sided students t-test, n=50,110 cells per group. ***p<0.001. Fig. 6g, images are representative for n=90 cells derived from 3 separate experiments. Fig. 6h, images are representative for n=90 cells derived from 3 independent experiments. Fig. 6i, images are representative for n=120 cells derived from 3 individual experiments. Fig. 6j, images are representative for n=120 cells derived from 3 separate experiments. Fig. 6k, images are representative for n=120 cells derived from 3 independent experiments. Fig. 6l, images are representative for n=120 cells derived from 3 separate experiments. Fig. 6m,n, images show

representative phenotypes of n=160 cells out of 4 autonomous experiments. Fig. 6o, image is a representative scheme derived from n=160 cells out of 4 autonomous experiments. Fig. 6p, images are representative for all indicated numbers of ECs analyzed in 6q-s, examined over 3 independent experiments. Fig. 6q-s, mean±s.e.m, unpaired two-sided students t-test, n=11,7,17,13,11 and 25 cells per indicated group. ns, not significant ***p<0.001.

Fig. 7a, experiment was carried out in triplicate, three independent times from each other resulting in n=9 measurement points per condition. Mean±s.e.m, unpaired two-sided students t-test, ***p<0.001. Fig. 7b, experiment was carried out in duplicate, three independent times from each other resulting in n=6 measurement points per condition. Mean±s.e.m, unpaired two-sided students t-test, **p=0.0063, ***p<0.001. Fig. 7c, experiment was carried out in duplicate, three independent times from each other, n=6 measurement points per condition. Mean ±s.e.m, unpaired two-sided students t-test. ns, not significant. Fig. 7d-f, images are representative for n=120 cells/condition derived from 4 independent experiments. Fig. 7g-i, images are representative for n=120 cells per condition examined over 4 independent experiments. Fig. 7j-l, images are representative for n=120 cells per condition derived from 4 independent experiments. Fig. 7m, mean±s.e.m, two-sided Mann Whitney U-test, n=69,26 and 22 cells per group derived from 4 autonomous experiments. ***p<0.001. Fig. 7n, determined were the cell-sizes of n=52 cells per condition in two separate experiments. Mean±s.e.m, unpaired two-sided students t-test. ns, not significant, ***p<0.001. Fig. 7o,p, images are representative for n=100 cells per condition derived from 3 independent experiments. Fig. 7q,r, images are representative for n=100 cells per condition derived from 3 independent experiments. Fig. 7s, mean±s.e.m, unpaired two-sided students t-test, n=7,9 cells per indicated group. ***p<0.001. Fig. 7t, images are representative for n=130 cells examined over 3 autonomous experiments. Fig. 7u, images are representative for n=130 cells examined over 3 autonomous experiments. Fig. 7v, images are representative for n=130 cells examined over 3 independent experiments. Fig. 7w, images are representative for n=130 cells examined over 3 independent experiments.

Fig. 8a,b, images are representative for n=80 aISVs per genotype examined over 4 separate experiments. Fig. 8c,d, images are representative for n=76,75 aISVs per genotype examined over 3 independent experiments. Fig. 8e, mean±s.e.m, two-sided Mann Whitney U-test, n=35 aISVs per genotype, ***p<0.001. Fig. 8f, mean±s.e.m, two-sided Mann Whitney U-test, n=34,48 aISVs per indicated genotype, ***p<0.001. Fig. 8g, mean±s.e.m, two-sided Mann Whitney U-test, n=51,70 aISVs/genotype. ns, not significant. Fig. 8h-k, images are representative for n=96,83,72,79 examined aISVs per genotype derived from 3 independent experiments. Fig. 8l-n, images are representative for n=116,103,112 examined aISVs per genotype. Fig. 8o, (left panel) mean±s.e.m, unpaired two-sided students t-test, n=18,19,13,14 aISVs per indicated genotype. ns, not significant; ***p<0.001; (right panel) mean±s.e.m, unpaired two-sided students t-test, n=21,24,15,12 cells per genotype. ns, not significant; ***p<0.001. Fig. 8p-s, images are representative for n=84,78,89,92 aISVs per condition. Fig. 8t, mean±s.e.m, unpaired two-sided students t-test, n=15,21,16 and 13 cells per indicated genotype, ***p<0.001. Fig. 8u, n=12,12,10,18 cells per indicated genotype, mean ±s.e.m. Fig. 8v,w, mean±s.e.m, unpaired two-sided students t-test, n=8,10,10 (v) and n=8,16,14 (w) embryos per group. *p<0.05, **p=0.0061.

Fig. 9a, mean±s.e.m, unpaired two-sided students t-test, n=8,9,8,10 embryos per group. **p=0.0011; ***p=0.0001. Fig. 9b, mean±s.e.m, unpaired two-sided students t-test, n=18,20,22,16 cells derived from 6 biologically independent embryos/group; ***p<0.001; **p=0.0055; *p=0.0100.

Randomization	Experimental groups were not pre-selected based on the genotype or phenotype and were thus randomized.
Blinding	Investigators were blinded to group allocation during data collection as well as quantification. Genotyping of embryos was performed afterwards.

Reporting for specific materials, systems and methods

We require information from authors about some types of materials, experimental systems and methods used in many studies. Here, indicate whether each material, system or method listed is relevant to your study. If you are not sure if a list item applies to your research, read the appropriate section before selecting a response.

Materials & experimental systems

n/a	Involved in the study
☐	☒ Antibodies
☐	☒ Eukaryotic cell lines
☒	☐ Palaeontology and archaeology
☐	☒ Animals and other organisms
☒	☐ Human research participants
☒	☐ Clinical data
☒	☐ Dual use research of concern

Methods

n/a	Involved in the study
☒	☐ ChIP-seq
☒	☐ Flow cytometry
☒	☐ MRI-based neuroimaging

Antibodies

Antibodies used	anti-alpha-tubulin primary antibody(clone DM1A) Sigma T6199; anti-beta-actin primary antibody (clone AC-40) Sigma A3853; anti-ENG primary antibody (3A9) Cell Signaling 14606; anti-HA primary antibody (HA.11) Covance MMS-101P; anti-integrin alpha5 primary antibody (clone EPR784) Abcam ab150361; anti-integrin beta1 primary antibody (clone P5D2) Abcam ab24693; anti-N-Cadherin primary antibody (3B9) Zymed 180224; anti-phospho-Paxillin primary antibody (pTyr118) Thermo Fisher Scientific 44722G; anti-pMLC-S19 primary antibody Cell Signaling 3675; anti-pMLC-T18S19 primary antibody Cell Signaling 3674; anti-Rac1 primary antibody (clone 102) BD Biosciences 610651; anti-Rac1B primary antibody MERCK 09-271; anti-RhoG primary antibody [1F3 B3 E5] Santacruz sc-80015; anti-spectrin (clone CT232) kind gift from Dr. Betty Eipper (UConne Health, Farmington, USA); doi: 10.1016/j.gene.2004.12.028; anti-Tiam1 [C-16] primary antibody Santacruz sc-872; anti-Trio primary antibody (clone D-20) Santacruz sc-6060; anti-Vav2 primary antibody [H-200] Santacruz sc-20803; anti-VE-Cadherin primary antibody (clone C-19) Santacruz sc-6458; anti-VE-Cadherin-Alexa Fluor 647 (clone 55-7H1) BD Biosciences 561567; anti-VEGFR2 primary antibody (55B11) Cell Signaling 2479;

donkey anti-goat IgG (H+L) DyLight-405 secondary antibody Jackson ImmunoResearch 705-475-147; donkey anti-mouse IgG (H+L) Alexa Fluor 568 secondary antibody Thermo Fisher Scientific A-10037; goat anti-mouse-HRP secondary antibody Dako P0447; goat anti-mouse IgG (H+L) Alexa Fluor 488 secondary antibody Invitrogen A-11029; rabbit anti-goat-HRP secondary antibody Dako P0449; swine anti-rabbit-HRP secondary antibody Dako P0399

Validation

Validation of antibodies can be found at https://www.antibodypedia.com/. Commerical antibodies were generated and validated by manufacturers (precise description given below). The Trio-spectrin antibody (clone CT232) was a kind gift of Dr. Betty Eipper (UConne Health, Farmington, USA). Validation of this antibody can be found in the study by McPherson et al, Gene 2005: https://www.sciencedirect.com/science/article/abs/pii/S0378111904007735?via%3Dihub.

In detail:

CD105/Endoglin (3A9) Mouse mAb #14606 has been validated by manufacturer Cell Signaling for usage in western blot and immunohistochemistry (paraffin) and has been used in Sugden et al, Nat Comm. 2017. Phospho-Myosin Light Chain 2 (Ser19) Mouse mAb #3675 has been validated by manufacturer Cell Signaling for application in western blot and immunofluorescence (immunocytochemistry) and has been used in Ando et al, JCB 2013. Phospho-Myosin Light Chain 2 (Thr18/Ser19) Antibody #3674 has been validated by manufacturer Cell Signaling for western blot and immunofluorescence (immunocytochemistry) and has been used in Ando et al, JCB 2013. Anti-α-Tubulin antibody, mouse monoclonal (mouse IgG1 isotype) (cat no T6199) is validated by manufacturer Sigma as an antibody specific for α-tubulin in immunoblotting assays and may be used for localization of α-tubulin in cultured cells or tissue sections. Monoclonal Anti-Actin (mouse IgG2a isotype, clone AC-40, cat no A3853) was validated by manufacturer Sigma as a monoclonal anti-actin antibody and was used for western blot analysis of Cos-7 cell lysates to ensure equal protein loading. Anti-HA (HA.11, MMS-101P) has been validated by manufacturer Covance for use in immunohistochemistry and was used in Kim Y, et al. 2016. Nat Commun. 7:10347. Recombinant Anti-Integrin alpha 5 antibody [EPR7854] (ab150361) has been validated by manufacturer Abcam for use in immunofluorescence and has been used in Gao et al, J Exp Med 2019. Anti-Integrin beta 1 antibody [P5D2] (ab24693) has been validated by manufacturer Abcam for use in immunofluorescence and has been used in Lee et al, Nat Comm. 2019. Anti-N-Cadherin (3B9) (180224) has been validated by manufacturer Zymed/Thermo Scientific for use in immunofluorescence and has been used in Timmerman et al, J Cell Sci 2013. Anti-phospho-Paxillin (pTyr118, 44722G) has been validated by manufacturer Thermo Scientific for use in immunofluorescence and has been used in Horton et al, JCB 2016. Purified Mouse Anti-Rac1 Clone 102/Rac1 has been validated by manufacturer BD Biosciences for use in western blot and has been used in Rijssel et al, MBC 2012. Anti-Rac1B (09-271) has been validated by manufacturer MERCK for use in western blot and has been used in Baker et al, Cancers 2020. Anti-RhoG [1F3 B3 E5] (sc-80015) has been validated by manufacturer Santacruz for use in western blot and has been used in Rijssel et al, MBC 2012. Anti-Tiam1 [C-16] (sc-872) has been validated by manufacturer Santacruz for use in immunofluorescence and has been used in Zhu et al, JBC 2014. Anti-Trio (clone D-20) (sc-6060) has been validated by manufacturer Santacruz for use in western blot and has been used in Rijssel et al, MBC 2012. Anti-Vav2 [H-200] (sc-20803) has been validated by manufacturer Santacruz for use in immunofluorescence and has been used in Rijssel et al, MBC 2012. Anti-VE-Cadherin (clone C-19) sc-6458 has been validated by manufacturer Santacruz for use in immunofluorescence and western blot and has been used in Rijssel et al, Biol Open 2013. Anti-VE-Cadherin-Alexa Fluor 647 (clone 55-7H1) has been validated by manufacturer BD Biosciences for use in immunofluorescence and has been used in Heemskerk et al, Nat Comm. 2015. Donkey anti- goat IgG (H+L) DyLight-405 (705-475-147) has been validated by manufacturer Jackson ImmunoResearch for use in immunofluorescence and has been used in Heemskerk et al, Nat Comm. 2015. Donkey anti- mouse IgG (H+L) Alexa Fluor 568 (A-10037) and goat anti- mouse IgG (H+L) Alexa Fluor 488 (A-11029) have been validated by manufacturer Thermo Scientific/Invitrogen for use in immunofluorescence and has been used in Heemskerk et al, Nat Comm. 2015. Goat anti mouse-HRP (P0447), rabbit anti goat-HRP (P0449), swine anti rabbit-HRP (P0399) have been validated by manufacturer Dako for use in western blot and have been used in Rijssel et al, MBC 2012.

Eukaryotic cell lines

Policy information about cell lines

Cell line source(s)	Primary Human Umbilical Vein Endothelial Cells (HUVECs, C2519A) and Primary Human Aortic Endothelial Cells (HAECs, CC-2535) were acquired from Lonza (Verviers, Belgium).<br>Primary Human Umbilical Artery Endothelial Cells (HUAECs) were isolated as described in van Geemen et al, ATVB 2014.
Authentication	Cells were authenticated by the manufacturer (Lonza, Verviers, Belgium) and were routinely checked by immunofluorescence, flow cytometry and western blot for expression of standard endothelial cell markers (VE-Cadherin, CD31, ICAM-1, VCAM-1, etc).
Mycoplasma contamination	All cell lines were frequently tested negative for mycoplasma contamination.
Commonly misidentified lines (See ICLAC register)	No commonly misidentified cell lines were used in this study.

Animals and other organisms

Policy information about studies involving animals; ARRIVE guidelines recommended for reporting animal research

Laboratory animals	The zebrafish (Danio rerio) was used as animal model. The wild type strains AB and TL were used for outcrossings. Mutant and transgenic lines were outcrossed to wild type fish after maximally two consecutive incrossings. New generations were established from embryos derived from several small group crosses. Both males and females of the following mutant and transgenic lines have been used:<br>gBAC(flt1:YFP)hu4624, Tg(flt1enh:tdTomato)hu5333, TgBAC(flt4:mCitrine)hu7135, Tg(kdrl:hsa.HRAS-mcherry)s916, Tg(fli1a:lifeactEGFP)mu240, Tg(fli1a:nEGFP)y7, Tg(fli1ep:gal4ff)ubs4, Tg(UAS:VE-cadherinΔC-EGFP)ubs12, Tg(mpeg1:GAL4-VP16)gl24, Tg(UAS:E1b:Kaede)s1999t, flt1ka601, flt1ka605, vhlhu2114, cdh5ubs8, nrp1ahu10012, flt4mu407, TgTm(flt1_E3_HAHA)ka611, Tg(flt1enh:sflt1_Δ7-HAHA)ka612, Tg(503unc:eGFP-p2A-plgf)ka613, Tg(503unc:eGFP-p2A-vegfba)ka614, Tg(flt1enh:GFP-TRION)ka615.<br>All embryos used for the analyses were derived from matings of zebrafish no older than two years.
Wild animals	No wild animals were used in this study.
Field-collected samples	No field-collected samples were used in this study.
Ethics oversight	Zebrafish husbandry and experimental procedures were approved by the government of Baden-Wuerttemberg, Regierungspraesidium Karlsruhe, Germany (Akz.: 35-9185.81/G-93/15; Akz.: 35-9185.81/G-93/19), the government of Berlin, Regierungspraesidium Berlin, Germany (Akz.: Reg0318/13) and the government of Nordrhein-Westfalen, Germany (Akz.: 81-02.05.40.19.044).

Note that full information on the approval of the study protocol must also be provided in the manuscript.
